# Improved Perovskite Solar Cells with an Environmentally Friendly Phthalocyanine Hole Extracting Interlayer

**DOI:** 10.1021/acsaem.5c03517

**Published:** 2026-02-26

**Authors:** Suresh K. Podapangi, Laura Mancini, Daimiota Takhellambam, Jie Xu, Luigi Angelo Castriotta, Giuseppe Mattioli, Venanzio Raglione, Federica Palmeri, Daniela Caschera, Anatoly P. Sobolev, Antonio Cricenti, David Becerril Rodriguez, Marco Luce, Aldo Di Carlo, Gloria Zanotti, Thomas M. Brown

**Affiliations:** ○ CHOSE (Centre for Hybrid and Organic Solar Energy), Department of Electronic Engineering, Tor Vergata University of Rome, 00133 Rome, Italy; ◆ Istituto di Struttura della Materia, Consiglio Nazionale delle Ricerche (ISM-CNR), via del Fosso del Cavaliere 100, Rome 00133, Italy; § Istituto di Struttura della Materia, Consiglio Nazionale delle Ricerche (ISM-CNR), Strada provinciale 35d/9, Montelibretti, Rome 00010, Italy; ∥ Department of Chemistry, La Sapienza University of Rome, P. le Aldo Moro 5, 00185 Rome, Italy; ⊥ Istituto per lo Studio dei Materiali Nanostrutturati (ISMN), Consiglio Nazionale delle Ricerche (CNR), Strada provinciale 35d/9, Montelibretti, Rome 00010, Italy; # Istituto per i Sistemi Biologici (ISB) Consiglio Nazionale delle Ricerche (CNR), Strada provinciale 35d/9, Montelibretti, Rome 00010,Italy

**Keywords:** Perovskite solar cells, zinc phthalocyanine, interlayer, pot-economical synthesis, indoor photovoltaics

## Abstract

We investigate the use of phthalocyanine, from the family of multipurpose functional organic complexes, as an interlayer between the hole-selective contact and the perovskite in self-assembled monolayer-based p-i-n perovskite solar cells. This phthalocyanine interlayer effectively mitigated recombination losses that were occurring between the self-assembled hole-extraction monolayer based on the carbazole functional group and the perovskite film. Furthermore, the crystallinity of the perovskite semiconductor was enhanced, which reduced nonradiative recombination and resulted in an increase in shunt resistance and a higher open-circuit voltage. The efficiency improved from 18.4% to 20.2%. A similar boost in efficiency was found under indoor lighting conditions (from 27.3% to 30.1%). The tetra-3,5-dimethylphenoxy-zinc phthalocyanine (DMPO4) molecule synthesized for this work also enhanced device stability under ISOS-D1 tests with the average *T*_80_ increasing from 1134 h to 1347 h with its incorporation. A purpose-designed synthetic strategy, yielding a total *E*-factor below 200, broadens the practical applicability of these versatile and cost-effective molecular materials.

## Graphical abstract

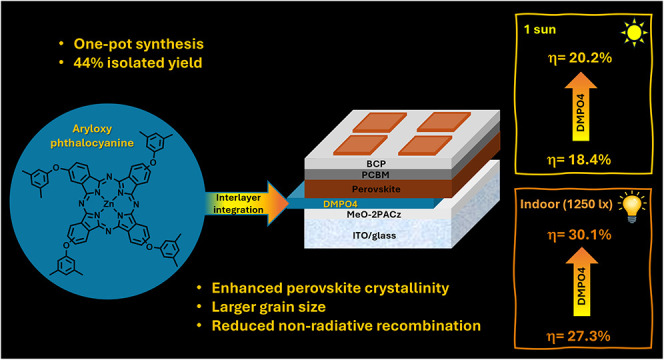

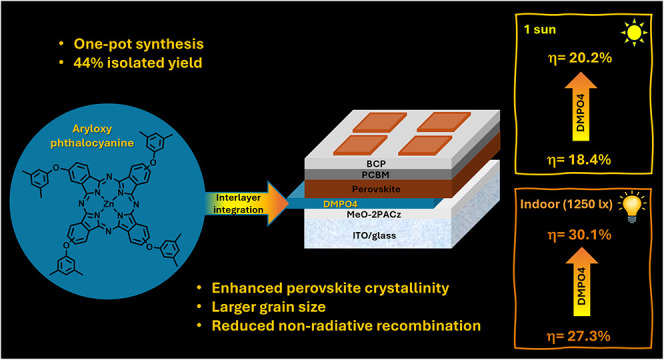

## 1 Introduction

Composition engineering, addition of passivating agents and other additives in the precursor inks, and dimensional engineering play a key role in developing perovskite cells with increasing power conversion efficiencies (PCEs) that have now approached those of silicon.^1–3^ The quality of the active absorber layer is crucial. However, the introduction of charge collection layers is equally critical for the efficient collection of carriers. Self-assembled monolayers (SAMs) have shown excellent hole collection capabilities and have proven beneficial to increase PCE due to their conformal coverage.^4^ Recombination losses are a major issue in reaching the target theoretical maximum efficiencies set by the Shockley - Queisser limit.^5^ Interfacial modification with interlayers has therefore become an important strategy in the fabrication of efficient multilayer stack architectures. Interlayers can passivate interfaces as well as the perovskite and/or charge transport layers to reduce recombination probabilities.^6–10^ This is especially important under low light illumination, typically found indoors under which conditions PSCs have shown great promise.^11–14^

Phthalocyanines are planar aromatic compounds with extended conjugation that have been successfully implemented as hole-transport materials delivering efficiencies above 20%,^15–20^ but in the past few years, they have also been investigated as interlayers in n-i-p architectures. Several derivatives have been deposited, for example, by immersion of the perovskite layer in a phthalocyanine solution^21^ or during the antisolvent step,^22,23^ with significant improvements of photovoltaic parameters and stability of the resulting devices. Aryloxy-substituted phthalocyanines possess high chemical and thermal stability, suitable hole mobilities, and appropriate energy levels alignments, even in dopant-free devices.^19,24^ In this work, we report the use of a newly synthesized tetra-3,5-dimethylphenoxy-zinc phthalocyanine (DMPO4) as an interlayer in p-i-n architecture deposited between the SAM and the perovskite active layer. We show significant improvement in both the efficiency and stability of solar cells. The molecule was obtained through a specially developed one-pot process rather than with the two-step protocol that is normally used in the literature for the synthesis of aryloxy-substituted phthalocyanines. The main advantage of this approach is the reduction in the overall time required to obtain the desired product and the amount of chemicals necessary for its purification. In this framework, the development of greener strategies, aimed at minimizing the use of toxic chemicals in the synthesis and processing of the materials for the final devices, represents an important research direction in the field of PSCs.^25–27^

## 2 Experimental Section

### 2.1 Materials and Methods

All reagents and solvents for the synthesis and characterization of DMPO4 were purchased from Merck Life Science S.r.l. (Milano, Italy), TCI Chemicals (Zwijndrecht, Belgium), and Carlo Erba Reagents (Cornaredo, Italy) and used without further purification. Reactions were purged and refilled three times, performed under argon, and monitored by thin-layer chromatography (TLC) employing a polyester layer coated with 250 mm F254 silica gel. Chromatographic filtrations were performed using silica gel 60A 35–70 μ. ^1^H and ^13^C NMR spectra as well as ^1^H–^1^H COSY, ^1^H–^13^C HSQC, and ^1^H–^13^C HMBC were recorded on a Bruker AVANCE 600 NMR spectrometer (Rheinstetten, Germany) operating at a proton frequency of 600.13 MHz in THF-*d*_8_; chemical shifts (δ) are given in ppm relative to the residual solvent peaks of the deuterated solvents. UV–vis spectra were recorded on a PerkinElmer Lambda 950 UV–vis/NIR spectrophotometer (PerkinElmer Italia, Milano, Italy) using dichloromethane (DCM) as a solvent. MALDI-TOF spectra were recorded at the Toscana Life Science facility on a MALDI-TOF/TOF Ultraflex III instrument (Bruker). Steady-state fluorescence spectra were recorded with a JobinYvon Fluorolog3 spectrofluorometer. The emissions were collected in the range of 420–780 nm, exciting the sample with a wavelength of 405 nm, and 5 nm grids. The corresponding excitation spectra have been collected in the range of 345–683 nm, under an excitation wavelength of 685 nm, with 2 nm grids. No filters were used. All experiments were performed at room temperature using quartz cuvettes with an optical path length of 10 mm. Time-resolved fluorescence measurements were carried out by a time-correlated single-photon counting (TCSPC) system (Horiba-Jobin Yvon), using a 405 nm pulsed laser diode and collecting the emission decay at the corresponding maximum emission wavelengths. The fluorescence decay profile was analyzed with decay analysis software (DAS6a HORIBA Scientific). Cyclic voltammetry (CV) investigations were carried out at 25 °C with a potentiostat-galvanostat Metrohm PGStat 204 in a conventional three-electrode cell equipped with a platinum disk (∼1 mm diameter) as the working electrode and a platinum wire as the auxiliary electrode. The reference electrode was Ag/AgCl, and the Fc^+^/Fc (ferrocenium/ferrocene) couple was used as external standard. The sample solutions were ∼10^–4^ M in freshly distilled dichloromethane, and dry tetra­(*n*-butyl)­ammonium tetrafluoborate (TBATFB) was used as the supporting electrolyte in a 0.1 M concentration. The solutions were previously purged for 10 min with nitrogen, and all measurements were performed under nitrogen. Voltammograms were recorded at a scan rate of 0.1 V s^–1^.

For solar cell fabrication, *N*,*N*-dimethylformamide (DMF; anhydrous-Sigma-Aldrich), dimethyl sulfoxide (DMSO; anhydrous-Sigma-Aldrich), toluene (Sigma-Aldrich), chlorobenzene (CB) (Sigma-Aldrich), isopropyl alcohol (IPA) (Sigma-Aldrich), 1,4-dichlorobenzene (DCB; Sigma-Aldrich), C_60_ Fullerene (C_60_; 99.50%, Solenne), [6,6]-phenyl-C61-butyric acid methyl ester (PCBM) (99.50%, Solenne), Bathocuproine (BCP; 96%, Sigma-Aldrich), Formamidinium iodide (FAI; 99.99%, Greatcell solar), Methylammonium bromide (MABr; 99.99%, Greatcell solar), Cesium iodide (CsI; 99.99%, Sigma-Aldrich), Lead Bromide (PbBr_2_; TCI), and Lead Iodide (PbI_2_; TCI) were purchased and used without further purification. Glass/ITO substrates (10 Ω sq^–1^) were purchased from Kintec.

### 2.2 Synthesis of DMPO4

In a two-necked 25 mL flask equipped with a reflux condenser, 0.388 g (3.18 mmol, 1.1 equiv) of 3,5-dimethylphenol and 0.880 g (5.78 mmol, 2 equiv) of 1,8-diazabicyclo(5.4.0)­undec-7-ene (DBU) were added, and the mixture was stirred in 4 mL of DMF for 20 min at room temperature. Then, 0.500 g (2.89 mmol, 1 equiv) of 4-nitrophthalonitrile was added, and the mixture was allowed to react at 60 °C for 3 h. After this, 0.171 g (0.78 mmol, 0.27 equiv) of zinc acetate dihydrate and 0.440 g (2.89 mmol, 1 equiv) of DBU were added to the mixture, which was stirred at 150 °C for 20 h. It was then cooled, treated with 3.5 mL of HCl 1.0 M, filtered, and washed with 5 mL of water. The resulting crude was purified by filtration on a silica pad (6 g) using 60 mL of a 5:1 (v/v) petroleum ether/tetrahydrofuran mixture (total volumes: 46.7 mL of petroleum ether (40–70 °C), 13.3 mL of THF). The resulting dark purple/blue solid was further purified by Soxhlet extraction using methanol as solvent (0.335 g, 0.32 mmol, 44%). ^1^H NMR (600 MHz, THF-*d*_8_) δ 9.13–9.04 (d, 4H, H-3), 8.75–8.69 (m, 4H, H-6), 7.76–7.73 (m, 4H, H-2), 7.08–7.03 (m, 8H, H-2′,6′), 6.96–6.93 (m, 4H, H-4′), 2.43–2.40 (m, 24H, CH_3_). ^13^C NMR (151 MHz, THF-*d*_8_) δ 160.60 (C-1), 158.84 (C-1′), 141.25 (C-5), 140.87–140.81 (C-3′,5′), 134.50–134.26 (C-4), 126.50 (C-4′), 124.82 (C-3), 121.45 (C-2), 118.41–118.23 (C-2′, 6′), 112.82 (C-6), 21.72 (CH_3_). UV–vis (DCM, nm) [log ε]: 675 [5.32], 350 [5.02]; MALDI-TOF (*m*/*z*) [M]^•+^: 1056.41. Elemental analysis: calcd C (72.62); H (4.57); N (10.59); found C (72.35); H (4.51); N (10.11).

### 2.3 Computational Methods

The most stable configuration of DMPO4 in dichloromethane solution was found using a conformer-rotamer search algorithm based on the GFN2-xTB tight-binding Hamiltonian.^28–30^ The lowest-energy configurations have been used as starting points for (time dependent) density functional theory studies, carried out using the ORCA suite of programs^31,32^ All simulations have been performed using a selection of exchange-correlation functionals optimized for properties calculations, together with the def2-TZVPP basis set, as discussed in more detail in the Supporting Information.

### 2.4 Fabrication of Perovskite Solar Cells

#### 2.4.1 SAM HTL Solution Preparation

(2-(3,6-Dimethoxy-9*H*-carbazol-9-yl)­ethyl)­phosphonic acid (MeO-2PACz) solution was prepared by adding 0.33 mg of MeO-2PACz in 1 mL of methoxyethanol. The solution was stirred overnight and used without any filtration.

#### 2.4.2 Interlayer Solution Preparation

The DMPO4 solution was prepared by stirring 5 mg of the material in 1 mL of DMF for 2 h at 75 °C, then letting it sit for 30 min, and filtering the solution before spin coating.

#### 2.4.3 Perovskite Ink Preparation

We use a triple cation perovskite (CSFAMA) with the composition Cs_0.05_MA_0.15_FA_0.8_Pb­(I_0.83_Br_0.17_)_3_ as reported in our previous work.^33^ The perovskite ink solution was prepared with a concentration of 1.38 M by mixing 0.05 mM CsI (17.56 mg), 0.15 mM MABr (23.75 mg), 0.8 mM FAI (187.92 mg), 0.82 mM PbI_2_ (532.86 mg, ∼2% excess), and 0.18 mM PbBr_2_ (91.47 mg) in 1 mL of a DMF:DMSO mixture (1:4 v/v). The solution was stirred overnight at room temperature and filtered through 0.45 μm PTFE filter before deposition.

#### 2.4.4 Device Fabrication

2.5 × 2.5 cm^2^ glass/ITO samples were patterned with a UV ns laser (Spectra physics, Andover, MA, USA) and diced with a glass cutter (Dyenamo, Stockholm, Sweden). Samples were scrubbed with water and soap solution and cleaned with three stages of ultrasonic bath for 10 min each: first in water and soap (Hellmanex 1% in deinonized water), then in ultrapure water, and finally in IPA. After drying, they were treated for 15 min in a UV–O_3_ instrument (Novasonic). Samples were immediately transferred to a nitrogen-filled glovebox. First, 120 μL of MeO-2PACz ink was dropped onto the substrate to ensure full coverage. After waiting for 3 s, the solution was spin-coated at 4000 rpm for 30 s and then annealed at 100 °C for 10 min. Once cooled, a DMPO4 solution was spin-coated at 4000 rpm for 20 s. Next, the perovskite ink was spin-coated at 4000 rpm for 30 s, and after 20 s, 150 μL of CB was dropped onto the spinning film. The film was then annealed at 100 °C for 10 min. For the electron transport layer, 27 mg/mL PCBM was dissolved in a 3:1 CB:DCB solvent mixture and stirred overnight. Then, 80 μL of the PCBM solution was dynamically dispensed onto the substrate and spin-coated at 1600 rpm for 35 s, followed by annealing at 100 °C for 5 min. Immediately after, 80 μL of BCP (0.5 mg/mL in IPA, stirred for two nights) was spin-coated at 4000 rpm for 35 s without additional drying. Finally, 100 nm of Cu was deposited via thermal evaporation using a shadow mask.

#### 2.4.5 Device Characterization

The top-view scanning electron microscopy (SEM) images were collected using a Hitachi SU8000 scanning electron microscope. Grains size was analyzed using Nano Measurer 1.2 ^34^ software. Perovskite films were deposited on basic glass/ITO/interlayer substrates to directly probe the perovskite surface morphology without interference from the transport layers or metal electrodes. The ultraviolet–visible (UV–vis) absorption spectra were performed on a UV–vis 2550 Spectrophotometer from Shimadzu.^35^ X-ray diffraction (XRD) measurements were performed in reflection mode on a Rigaku SmartLab diffractometer by means of Kα fluorescence lines (Kα_1_ [Å] = 1.54060; Kα_2_ [Å] = 1.54441) of a Cu anode. XRD measurements were collected in Bragg–Brentano configuration for 2θ ranges from 5° to 45° focusing the impinging beam with fixed divergent slits (1/4–1/2°). A solid-state hybrid PIXcel3D detector, working in 1D linear mode, accomplished the detection, and continuous scan mode was adopted. Current density–Voltage (*J*–*V*) curves under one sun illumination were measured by a source meter (Keithley 2400) under a calibrated solar simulator (ABET Sun 2000, class A) providing standard test conditions (AM1.5G, 1000 W/m^2^) at room temperature. For indoor *J*–*V* measurements, a white LED (Osram Parathom Classic P25) was used as the light source, and the light intensity was calibrated to obtained specific illuminance (e.g., 200, 500, and 1000 lx) by a luxmeter (NIST-traceable calibrated Digisense 20250-00). More details can be found in our previous works.^34,36,37^ The *J*–*V* curve of each device was obtained by masking the active area (0.09 cm^2^) with a mask. Time-resolved PL (TRPL), dark current density versus voltage (Dark *J*–*V*), light intensity dependence of open circuit voltage (*V*_OC_), and transient photovoltage (TPV) decay were collected by using a modular testing platform (Arkeo-Cicci Research s.r.l.).^34,37^ A homemade Kelvin Probe system was used to determine the sample work function. The probe was driven by a piezoelectric actuator, employing a 2 × 1.5 mm^2^ gold mesh as the reference electrode. For surface electronic structure characterization, X-ray and ultraviolet photoelectron spectroscopy (XPS/UPS) were conducted in a Vacuum Generator VG-450 UHV chamber equipped with an Al Kα (1486.6 eV) source, achieving an energy resolution of ∼0.1 eV.

## 3 Results and Discussion

### 3.1 Synthesis of DMPO4

The synthesis of tetra-aryloxy-substituted metallophthalocyanines commonly consists of two steps: (i) the insertion of the desired aryloxy group on the phthalonitrile ring via nucleophilic aromatic substitution (S_N_Ar) and (ii) its tetramerization in a high-boiling solvent such as dimethylaminoethanol (DMAE), *n*-pentanol or *N*,*N*-dimethylformamide, aided by an organic base like DBU and a templating salt.^38–42^ Consequently, two workups, including purification processes, are required to obtain the final product. In contrast, our streamlined approach for the synthesis of DMPO4 takes advantage of the compatibility between the nucleophilic aromatic substitution (S_N_Ar) environment and the phthalocyanine ring formation conditions. We investigated the feasibility of performing both reactions sequentially in the same reaction flask, which resulted in a more efficient, one-pot process. We screened different reaction conditions by varying several parameters such as the base (DBU or potassium carbonate), reagent molar ratios, and reaction temperature throughout the process as summarized in Table S1 in the Supporting Information. We found that the optimal reaction conditions were achieved with a phthalonitrile/phenol ratio of 1:1.10 and DBU as the base when the S_N_Ar reaction was conducted at a temperature of 60 °C. According to our experiments, the superior performance of DBU as a base under these conditions can be attributed not only to its higher basicity but also to its liquid state, allowing for homogeneous dispersion in the reaction medium. The further addition of DBU in the second step was found to enhance the phthalocyanine formation. DMPO4 was then straightforwardly purified by filtration on a short silica pad followed by Soxhlet extraction of impurities in methanol, affording a 44% isolated yield calculated with respect to the limiting reagent after purification. The synthetic pathway is reported in Figure 1a.

**Figure 1:**
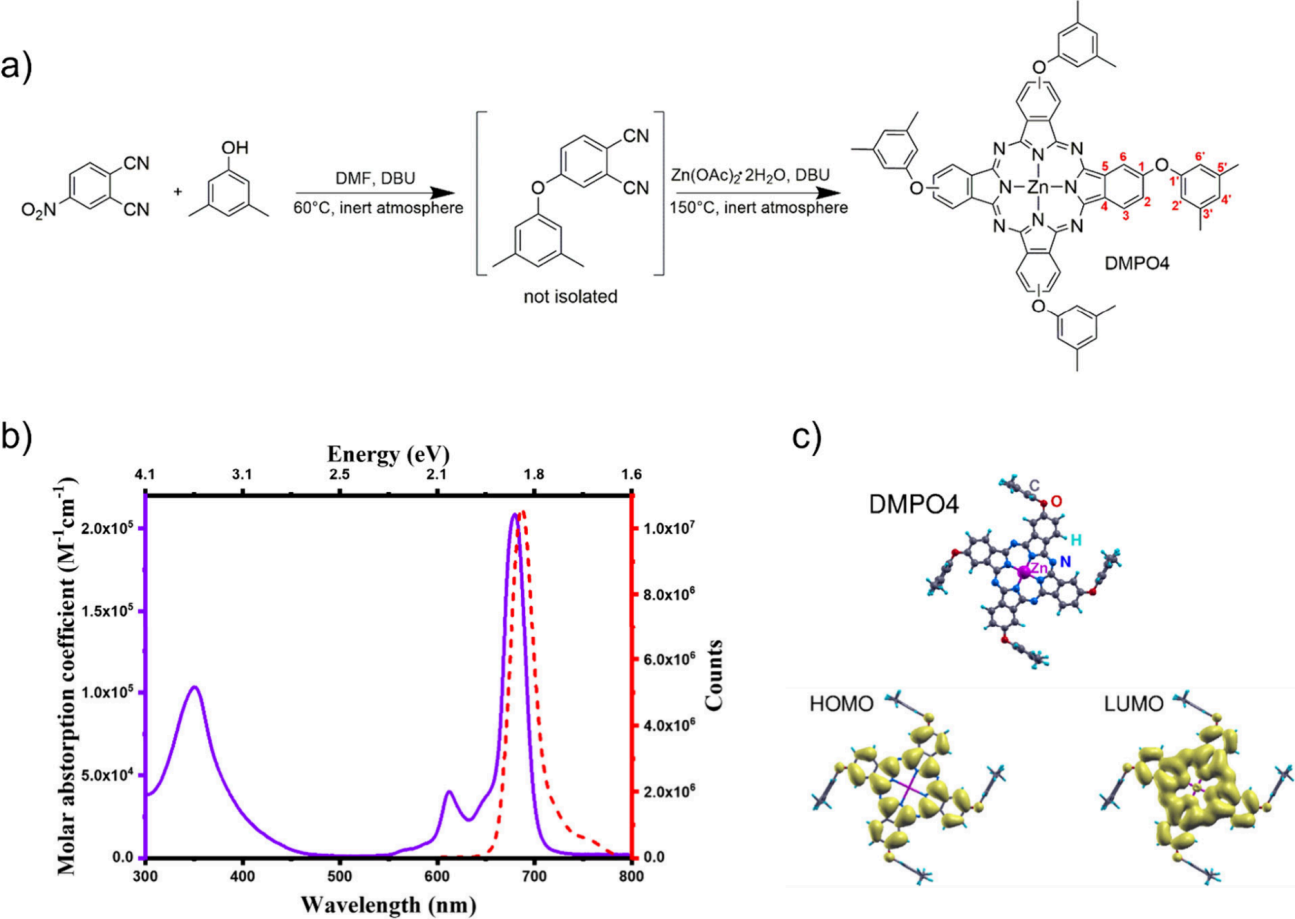
(a) Synthesis of tetra-3,5-dimethylphenoxy-zinc phthalocyanine (DMPO4). The optimal reaction conditions are indicated by the reaction arrows. Red numbers label the carbon atoms according to the ^13^C NMR peak assignation. (b) Normalized ground state electronic absorption (solid line) and fluorescence emission (dashed line) of DMPO4 in dichloromethane. (c) Optimized geometry and |ψ|^2^ of frontier orbitals of DMPO4, calculated using DFT methods at the def2-TZVPP@B3LYP level of theory.

The NMR spectra provided in the Supporting Information as Figures S1 to S5 and the MALDI-TOF mass spectrum reported as Figure S6, together with elemental analysis, confirmed the chemical nature of the derivative. Its good solubility in a wide variety of solvents like THF, acetone, chlorobenzene, and anisole makes it suitable for solution-processing in optoelectronic devices. ^1^H NMR investigation provides the expected multiplets for the aromatic protons that appear in the range of 6.91–9.13 ppm. The complexity of the methyl signal centered at 2.41 ppm suggests that DMPO4 is formed as a statistical mixture of regioisomers having *C*_4h_, C_2v_, *D*_2h_, and *C*_s_ molecular symmetry, a common feature of tetra-substituted phthalocyanines that generally does not affect their electronic properties.^43^ The optical properties of DMPO4 in solution were characterized using UV–vis and fluorescence spectroscopy, provided in the Supporting Information as Figures S7–S8. Figure 1b reports the ground state electronic absorption spectrum of DMPO4 in dichloromethane and the steady state emission decay. The main absorption signals, the Q and Soret bands, peak at 679 and 350 nm, respectively, and their narrowness suggests the absence of aggregated species. The steady-state emission maximum lies at 688 nm, and the resulting Stokes shift is 193 cm^–1^, suggesting small structural changes between ground and excited states as usually seen in pristine and substituted phthalocyanines. An optical band gap (*E*_opt_) of 1.82 eV was estimated from the intersection of the normalized absorption and emission spectra in close agreement with a theoretical estimate of 1.81 eV (Table S2). Time-correlated single photon counting measurements, shown in Figure S8, have been monoexponentially fit to obtain *t*_1_ = 3.02 ns. Cyclovoltammetric measurements in dichloromethane have been performed vs the Fc^+^/Fc redox couple and show quasi-reversible first oxidation (0.190 V) and reduction (−1.394 V) processes (Figure S9) resulting in −5.28 eV and −3.69 eV electrochemical potentials versus vacuum, respectively, in nice agreement with theoretical estimates of −5.21 eV and −3.42 eV, respectively (Table S2). These values, with respect to those of a perovskite semiconductor, are conducive to the transport of holes and the blocking of electrons when incorporated in a heterostructure. The optoelectrochemical parameters of the synthesized phthalocyanine are summarized in Table 1.

**Table 1:** Optoelectrochemical Characterization of DMPO4 in Dichloromethane^a^

Molecule	λ_abs_ (nm) [log ε]	λ_em_ ^a^ (nm)	Stokes shift (cm^–1^)	*E*_opt_ (eV)	*E*_ox_ ^b^ (eV)	*E*_red_ ^c^ (eV)	Δ*E*_g_ ^d^ (eV)
DMPO4	675 [5.32]	688	192.7	1.82	–5.28	–3.69	1.59

a λ_abs_ = wavelength of maximum absorption; log ε = molar extinction coefficient (logarithmic scale); λ_em_ = wavelength of maximum emission; Stokes shift = energy difference between absorption and emission maxima; *E*_opt_ = energy derived from the absorption onset; *E*_ox_ = oxidation potential; *E*_red_ = reduction potential; Δ*E*_g_ = electrochemical band gap determined from *E*_ox_ – *E*_red_.

b Excitation wavelength: 685 nm.

c *E*_ox_ = −(*E*_1/2ox_ + 5.088) (eV).

d *E*_red_ = −(*E*_1/2red_ + 5.088) (eV).

e *E*_ox_ – *E*_red_.

### 3.2 DFT Simulations

Theoretical simulations support the experimental characterization of DMPO4. Electrochemical and optical features calculated using dichloromethane as an implicit solvent closely reproduce measurements (Table S2). The four dimethylphenoxy substituents attached to the archetypal phthalocyanine structure keep the benzene planes orthogonal to the macrocyclic plane (see Figure 1c), hindering the aggregation of molecules in stacks reported instead in the case of other coplanar aromatic substituents.^43^ Regarding electronic properties, the orthogonality between aryloxy substituents and the molecular core is responsible for their surprisingly weak electron donating effect, evidenced by the negligible delocalization of the frontier orbitals on such substituents, comparable to that reported in the case of *tert*-butyl substituents.^43,44^ A close comparison between the properties of DMPO4 and zinc tetra *tert*-butylphthalocyanine (ZnTTB) is reported in Table S2 and Figure S10 in the Supporting Information.

### 3.3 *E*-Factor and Cost Analysis

To evaluate the environmental sustainability of our synthesis in terms of consumption of materials, we calculated the *E*-factor, a relevant green chemistry metric defined as the ratio between the mass of waste and product obtained. In our case, considering all of the chemicals used including water, we obtained an *E*-factor of 196.1. Omitting the latter, the value is 181.2. This value is in line with or lower than those calculated for tetrasubstituted derivatives synthesized with a single step procedure.^35,45,46^ To evaluate the fabrication costs of DMPO4, we performed a cost-per-gram estimation according to a paper published in 2013 by Osedach et al.^47^ and reported in Table 2. The estimated price of 10.65 EUR/g (energy, facility maintenance and personnel costs, taxes and other charges excluded) makes the molecule effectively competitive, compared with the panorama of phthalocyanines for perovskite-based photovoltaics currently present in the literature.^35,48^ Table 2 also shows that the materials needed for the workup and purification steps have the greatest impact on the total cost. Complete information and detailed calculations are provided in the Supporting Information, Figure S11 and Table S3.

**Table 2:** Cost Estimation of DMPO4, Expressed in EUR/g

Molecule	Reactants	Reaction solvent	Workup/purification	Total
DMPO4	2.87 €/g	0.34 €/g	7.44 €/g	10.65 €/g

### 3.4 Performance of Perovskite Solar Cells

As shown in Figure 2, we utilized a DMPO4 molecule as an interlayer in triple cation perovskite (CSFAMA) solar cells with the following p-i-n structure: ITO/MeO-2PACz/­DMPO4/CsFAMA/­PCBM/BCP/Cu. We characterized the cells both at standard test conditions (1 SUN) and under indoor lighting.

**Figure 2:**
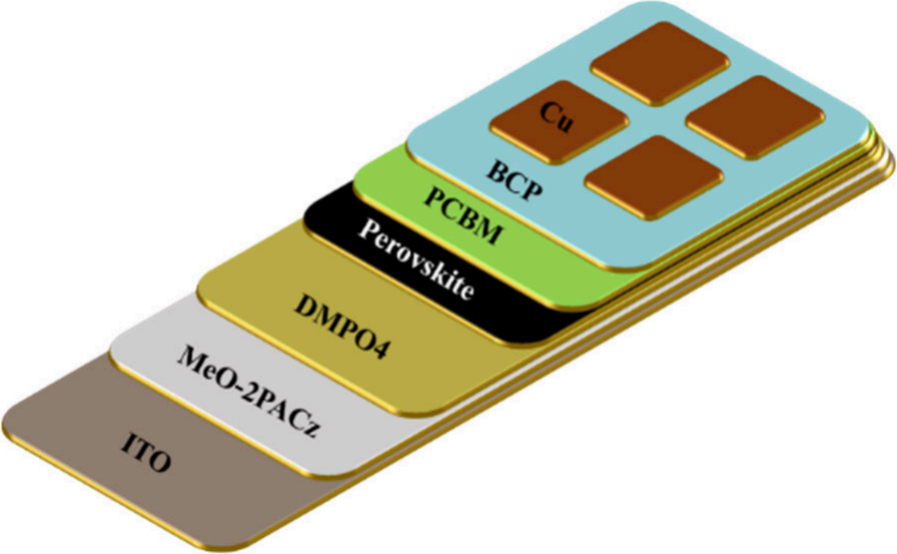
Schematic representation of the DMPO4 molecule as an interlayer in inverted perovskite solar cells (ITO/MeO-2PACz/­DMPO4/CsFAMA/­PCBM/BCP/Cu).

Figure 3 presents statistical box plots of the key photovoltaic parameters of perovskite solar cells incorporating a DMPO4 interlayer and reference devices measured under standard test conditions (STC). As shown in Figure 3a, devices with the DMPO4 interlayer exhibit a clear increase in power conversion efficiency (PCE) compared with the reference cells, accompanied by a narrower distribution, indicating improved device reproducibility. The enhancement in the PCE originates from concurrent improvements in the fill factor (Figure 3b), short-circuit current density (Figure 3c), and open-circuit voltage (Figure 3d). In particular, the DMPO4-based devices show higher average FF and *J*_SC_ together with a noticeable increase in *V*_OC_. Overall, the statistical analysis highlights the beneficial role of the DMPO4 interlayer in simultaneously enhancing device performance and operational consistency.

**Figure 3:**
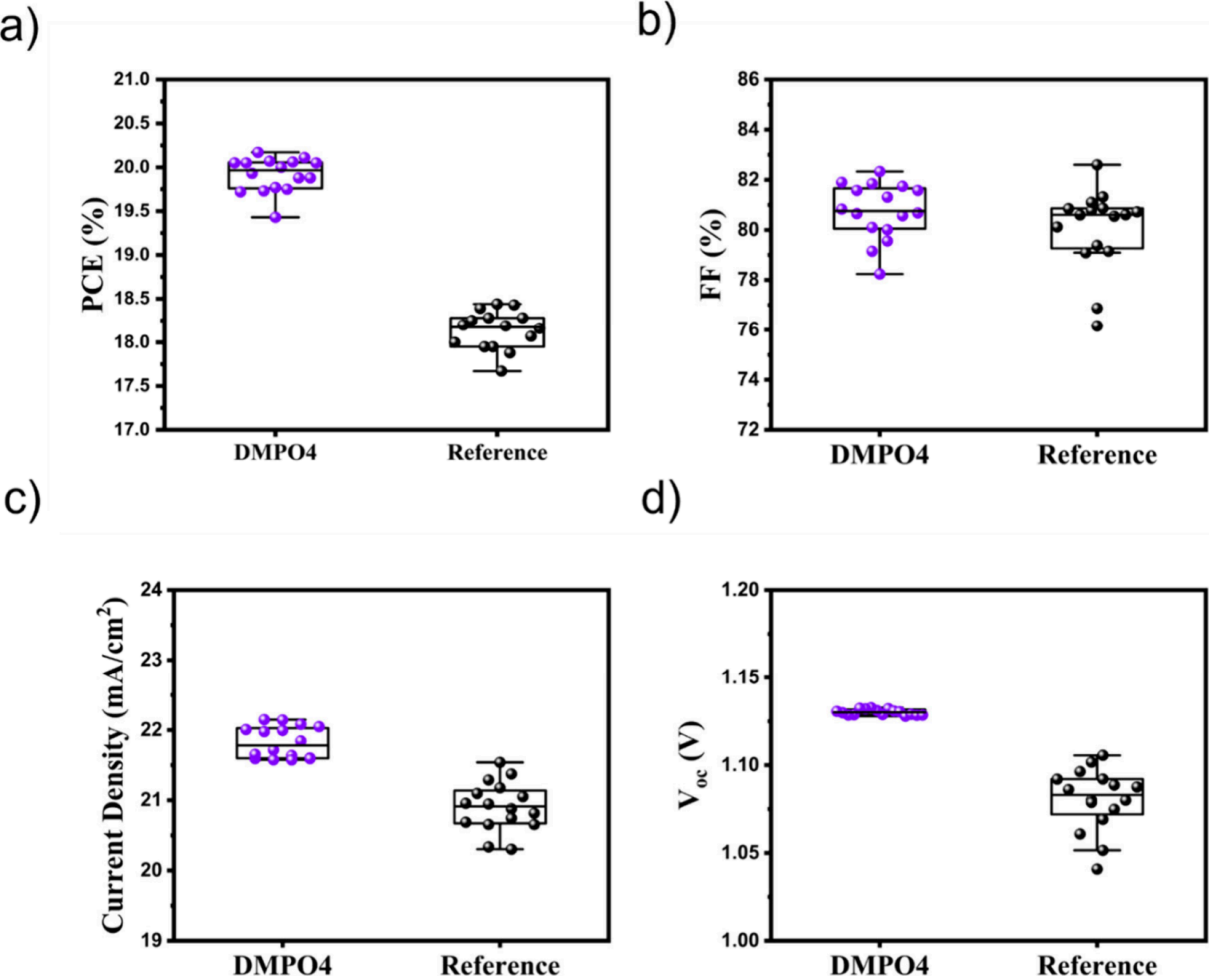
Photovoltaic parameters of perovskite solar cells with a DMPO4 interlayer (ITO/MeO-2PACz/­DMPO4/CsFAMA/­PCBM/BCP/Cu) and reference devices without DMPO4, measured under standard test conditions (STCs) (25 °C AM1.5G spectrum: 1000 W/m^2^). Box-and-whisker plots compare (a) power conversion efficiency (PCE), (b) fill factor (FF), (c) short-circuit current density (*J*_SC_), and (d) open-circuit voltage (*V*_OC_) for DMPO4-based devices and reference cells.

In Figure 4, we reported the *J*–*V* curves of the best ITO/MeO-2PACz/­DMPO4/CsFAMA/­PCBM/BCP/Cu devices under STC. The reference device, without a DMPO4 interfacial layer, delivered a PCE of 18.4% with an open-circuit voltage (*V*_OC_) of 1.11 V, a short-circuit current density (*J*_SC_) of 21.5 mA/cm^2^, and a fill factor (FF) of 81.3% (see Table 3). The device incorporating the DMPO4 interfacial layer exhibited enhanced performance with a PCE of 20.2%, *V*_OC_ of 1.13 V, *J*_SC_ of 22.1 mA/cm^2^, and a FF of 82.3%. We also analyzed the hysteresis factor (HF) of the devices, which quantifies the instability in the device’s performance during forward and reverse scans. The reference device exhibited a 2% hysteresis factor while the DMPO4-based device displayed no hysteresis (∼0%), demonstrating that the interlayer reduces interfacial defect concentrations. *J*_SC_ calculated from the EQE spectra was found to be consistent within the experimental error (5%) for both the reference and DMPO4 devices (See Figure S12 in the Supporting Information).

**Table 3:** Photovoltaic Parameters of Perovskite Solar Cells with a DMPO4 Interlayer (ITO/MeO-2PACz/DMPO4/CsFAMA/PCBM/BCP/Cu) and Reference Devices without an Interlayer^a^

Device name	Current density (*J*_SC_) (mA/cm^2^)	Voltage (*V*_OC_) (V)	Fill factor (FF) (%)	PCE (η) (%)
DMPO4	21.82 ± 0.22 (22.14)	1.13 ± < 0.01 (1.13)	80.75 ± 1.12 (82.33)	19.91 ± 0.19 (20.17)
Reference	20.90 ± 0.35 (21.53)	1.08 ± 0.01 (1.11)	80.04 ± 1.63 (81.32)	18.07 ± 0.34 (18.44)

a Measured under standard test conditions (AM1.5G, 1000 W/m^2^).

**Figure 4:**
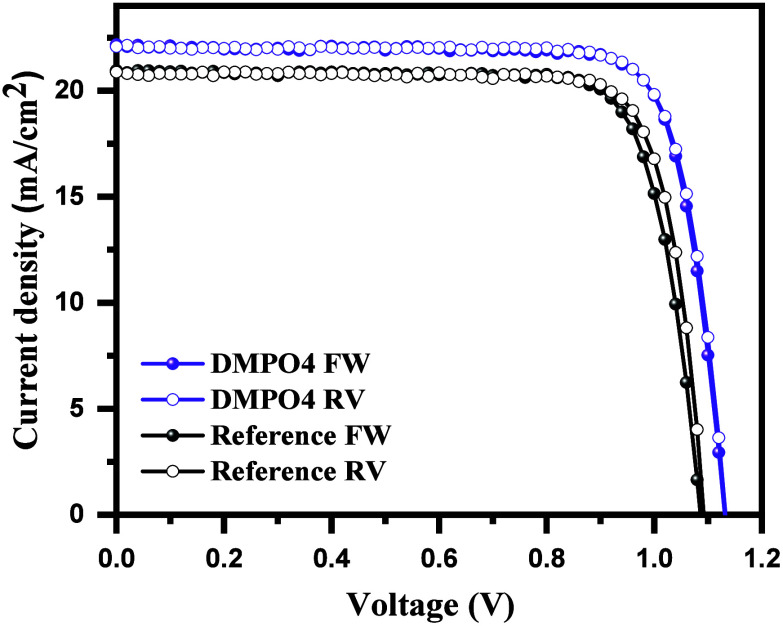
Current density–voltage (*J*–*V*) of the best glass/ITO/SAM/­FAMACs/HTM/­Cu cells with the reference device (Black spherical line) and DMPO4 interlayer device (Violet spherical line) measured under standard test conditions (STC, AM1.5G, 1000 W/m^2^).

The *J*–*V* characteristic curve shown in Figure 5 represents the performance of a solar cell under indoor lighting conditions at two different illumination levels: 1250 and 250 lx. As expected, the current density is higher at 1250 lx compared to that at 250 lx, as increased illumination generates more charge carriers within the device. At 1250 lx under artificial light (white LED lamp OSRAM P25), the interlayer was also beneficial. It delivered a stabilized PCE of 30.1% compared to 27.3% of the reference device (Figure 5a). The *J*_SC_ values measured from the *J*–*V* scans were within 10% of the value of the integrated *J*_SC_ calculated from the EQE curve and irradiance values of LED at 1250 lx. Under one sun illumination, the short-circuit current density obtained from *J*–*V* measurements was 22.10 mA cm^–2^ for the DMPO4 device and 21.50 mA cm^–2^ for the reference device. The corresponding calculated *J*_SC_ values determined by integrating the EQE spectra were 21.48 mA cm^–2^ (DMPO4) and 21.50 mA cm^–2^ (reference), very close to the measured ones demonstrating the reliability: within 2.9% for the DMPO4 device and 0.02% for the reference. At 1250 lx LED illumination, the measured *J*_SC_ from *J*–*V* scans was 0.152 mA cm^–2^ (DMPO4) and 0.146 mA cm^–2^ (reference), both within 10% of those derived from the calculated *J*_SC_ integrating the EQE, confirming the reliability of the measurements.

**Figure 5:**
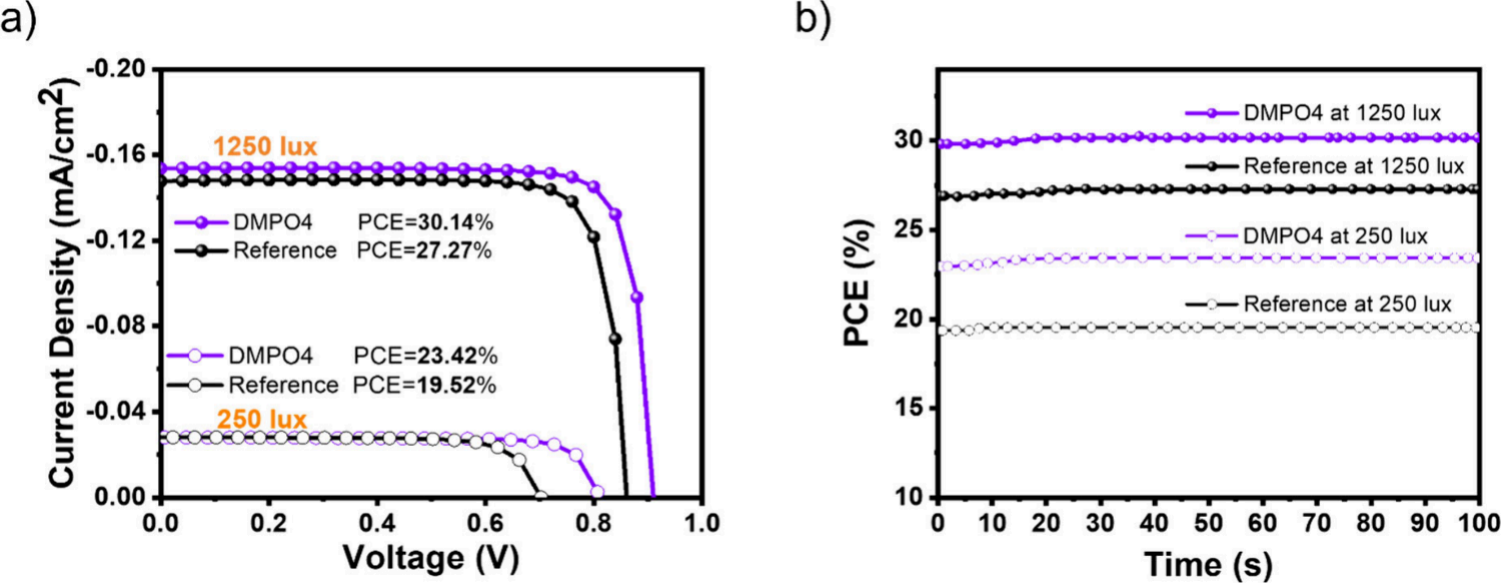
(a) Indoor current density–voltage (*J*–*V*) curves and (b) maximum power point tracking (MPPT) for the best-performing devices with DMPO4 (ITO/MeO-2PACz/­DMPO4/CsFAMA/­PCBM/BCP/Cu) and reference devices without DMPO4 measured under LED lamps at 250 and 1250 lx.

Under indoor conditions, a substantial fraction of the efficiency improvement resulting from the introduction of the interlayer can be attributed to *V*_OC_. The PCE increases from 27.27% to 30.14% (a relative gain of ∼10.5%), while *V*_OC_ improves from 0.86 to 0.915 V (a relative gain of ∼6.4%), suggesting that approximately half of the overall efficiency enhancement is due to *V*_OC_. A significant enhancement of *V*_OC_ indicates lower recombination losses and reduced reverse saturation current densities (*J*_0_).^49–51^ This reduction in recombination losses enhances overall energy conversion efficiency, making the interlayer a key factor in optimizing photovoltaic performance in low-light indoor environments. In fact, when incorporating DMPO4 as an interlayer, we observed a significant decrease in the reverse saturation current density (from 6.49 × 10^–5^ mA/cm^2^ to 3.39 × 10^–5^ mA/cm^2^ as shown in Figure 6a). The lowering of recombination is evident from the analysis of the shunt resistance (*R*_sh_) in the solar cells, with the DMPO4 interlayer device exhibiting a significantly higher *R*_sh_ (11.11 × 10^3^ Ω) compared to the reference device (9.24 × 10^3^ Ω).

**Figure 6:**
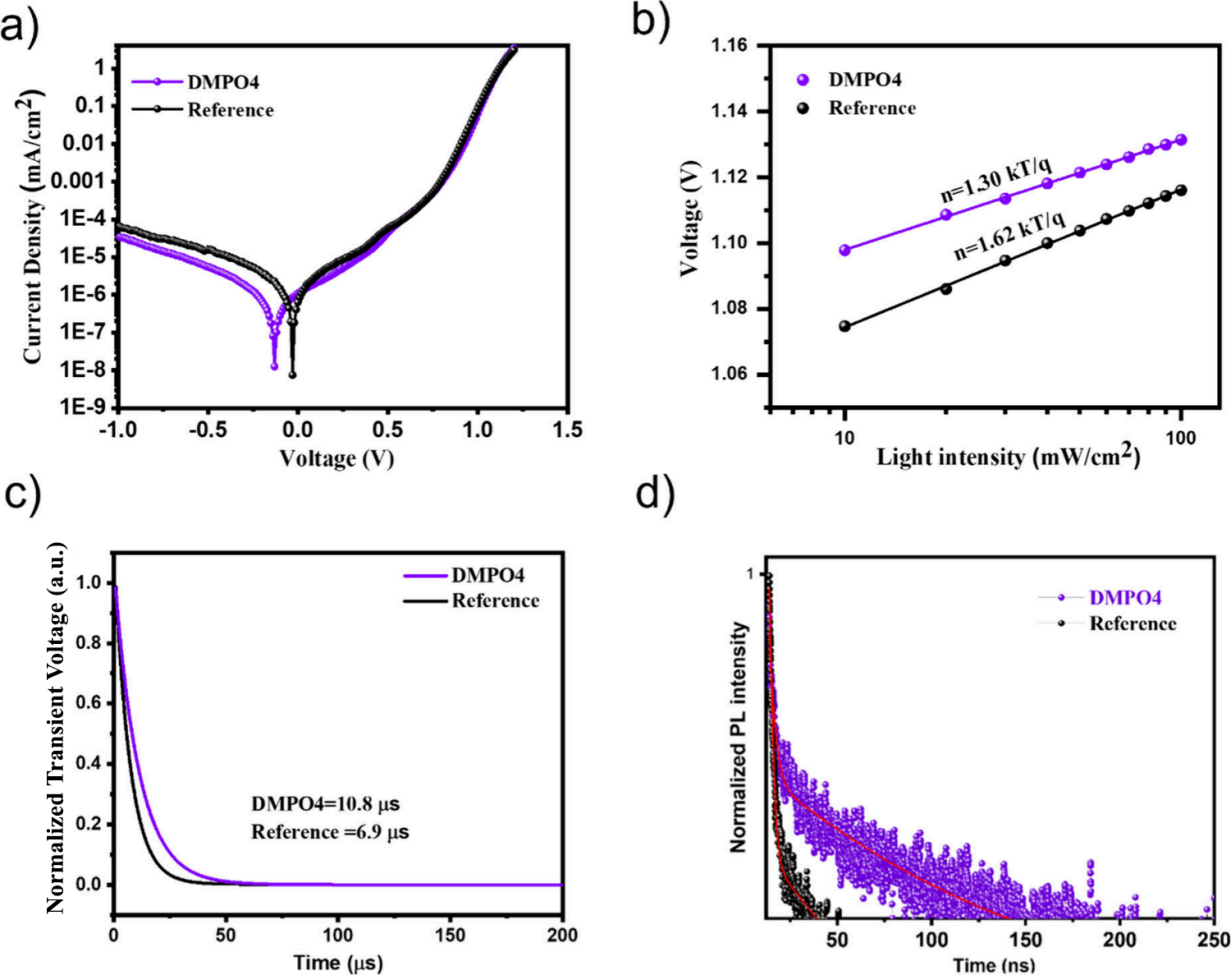
(a) Dark current–voltage characteristics of reference and DMPO4 devices plotted on a semilog scale; (b) light intensity dependence of *V*_OC_ for the calculation of ideality factors; (c) transient photovoltage (TPV) of perovskite solar cells with and without the DMPO4 interlayer, and (d) time-resolved PL (TRPL) decay spectra of perovskite films with and without the DMPO4 interlayer.

In Figure 6b, we explore the correlation between *V*_OC_ (open-circuit voltage) and light intensity, aiming to determine the ideality factor *n* (1 < *n* < 2), which is calculated using the following equation:

$$n=\frac{q}{kT}\frac{d\;V_{OC}}{d\;\ln\left(\phi \right)}$$

The dominant recombination mechanism of the devices can be identified: when the ideality factor approaches 1, it indicates that recombination mainly comes from free electrons and holes; on the other hand, when the ideality factor is closer to 2, it suggests that trap-assisted Shockley–Read–Hall (SRH) recombination becomes dominant, leading to decreased device efficiency.^12,52^ By performing the linear fitting of *V*_OC_/ln­(ϕ), we can calculate the ideality factor for both DMPO4 and the reference device. The value of *n* was 1.30 for the device with a DMPO4 interlayer and 1.62 for the reference device, indicating a lower recombination through defects confirming that the role of DMPO4 is significantly improving the quality of the interface.^53^ At the low optical power found under indoor illumination, keeping the recombination currents low is even more crucial to obtain high performance.^36^ The ratio between ON current (at +1 V) and OFF current (at −1 V) (*J*_ON_/*J*_OFF_) measured in the dark should be greater than 10^2^ to achieve high efficiencies under indoor illumination.^54^ The *J*_ON_/*J*_OFF_ ratios of the DMPO4 and reference devices were 1.89 × 10^3^ and 1.36 × 10^3^, respectively (Table 4). We extracted the *J*_ON_/*J*_OFF_ ratios from the dark *J*–*V* plot shown in Figure 6a. Both are high values, with the DMPO4 devices exhibiting a 39% higher ratio, consistent with the improvement in performance at these low illumination levels.^55^

**Table 4:** Ratio between Forward and Reverse Current (*J*_ON_/*J*_OFF_) and Shunt Resistances of DMPO4 and Reference Devices^a^

Device name	*J*_ON_ (mAcm^–2^)	*J*_OFF_ (mAcm^–2^)	*J*_ON_/*J*_OFF_	*R*_sh_ (Ω)
DMPO4	6.40 × 10^–2^	3.39 × 10^–5^	1.89 × 10^3^	11.11 × 10^3^
Reference	8.84 × 10^–2^	6.49 × 10^–5^	1.36 × 10^3^	9.24 × 10^3^

a *J*_ON_/*J*_OFF_ current ratio extracted from dark *J*–*V* measurements at +1 V (*J*_ON_) and −1 V (*J*_OFF_) shown in Figure 6a and shunt resistance (*R*_sh_) from *J*–*V* curves measured at STC.

The process of recombination can be studied using transient photovoltage (TPV) measurements where, when the incident light is turned off, voltage decreases due to the recombination of electrons and holes. Figure 6c shows the time constant extracted from a single exponential fit of the voltage decay profiles calculated under different low-light intensities.^56^ The reference device shows a shorter decay time of 6.9 μs, while in DMPO4 devices, a longer decay time of 10.8 μs is observed, indicating an extended carrier recombination time. In Figure 6d, the time-resolved photoluminescence (TRPL) study of the perovskite films revealed efficient charge extraction at the perovskite/MeO-2PACz interface. When the results are compared, the reference film displayed a biexponential decay^35,57^ with fast (1.50 ns) and slow (68.05 ns) components, while a different biexponential decay pattern (2.52 and 83.39 ns) was observed with the DMPO4 interlayer (Table 5). Again, the presence of the DMPO4 interlayer reduced nonradiative recombination processes at the perovskite/MeO-2PACz interface.

**Table 5:** Summary of the Fitting Parameters for the TRPL Decay Data^a^

Device name	A1	τ1 (ns)	A2	τ2 (ns)
DMPO4	0.88	2.54	0.26	83.39
Reference	0.13	1.50	0.20	68.05

a A1 and A2 represent the weighting factors of the τ1-fast decay component recombination via defect trapping and of the τ2-slow decay component associated with radiative recombination, respectively.

To quantify the defect-state density at the HTL/perovskite interface, hole-only devices with the architecture ITO/MeO-2PACz/DMPO4/perovskite/PTAA/Cu and corresponding reference devices without the DMPO4 interlayer were fabricated and characterized by using space-charge-limited current (SCLC) dark *J*–*V* measurements. The trap-state density (*N*_trap_) was extracted from the trap-filled limit voltage (*V*_TFL_) following previously reported methods:^58,35^

$$N_{\mathrm{trap}}=\frac{2V_{\mathrm{TFL}}\varepsilon_{0}\varepsilon }{eL^{2}}$$

where ε_0_ = 8.8 × 10^–12^ F m^–1^ is the vacuum permittivity, ε = 62.23 is the relative dielectric constant of the perovskite,^59^ *e* = 1.6 × 10^–19^ C is the elementary charge, and *L* = 450 nm is the perovskite thickness. Since all parameters except *V*_TFL_ are identical for both devices, variations in *N*_trap_ directly reflect differences in interfacial trap density.

From the SCLC curves shown in Figure S14, the reference device exhibits a *V*_TFL_ of 0.54 V, corresponding to a trap density of 1.82 × 10^16^ cm^–3^. In contrast, the device incorporating the DMPO4 interlayer shows a reduced *V*_TFL_ of 0.39 V, yielding a lower trap density of 1.32 × 10^16^ cm^–3^. This ∼27% reduction in *N*_trap_ confirms that the DMPO4 interlayer effectively passivates defect states at the HTL/perovskite interface. The lower trap density derived from SCLC analysis indicates suppressed trap-assisted nonradiative recombination and more efficient hole transport across the interface, consistent with the improved photovoltaic performance observed in DMPO4-based devices. Taken together, the SCLC, TPV, and dark *J*–*V* analyses provide a consistent mechanistic picture for the enhanced open-circuit voltage observed in DMPO4-based devices.

The reduced interfacial trap density induced by the DMPO4 interlayer suppresses trap-assisted recombination at the HTL/perovskite interface, thereby lowering recombination losses and leading to increased *V*_OC_.

To investigate the effect of the DMPO4 interlayer on the electronic structure of the perovskite films, XPS, UPS, and Kelvin probe measurements were performed. The X-ray photoelectron spectroscopy (XPS) spectra of the Pb 4f region (see Figure S15a in the Supporting Information) exhibit two distinct peaks at approximately 137.6 and 142.5 eV, corresponding to the Pb 4f_5_/_2_ and Pb 4f_7_/_2_ spin–orbit components, respectively. The observed spin–orbit splitting (∼5 eV) and binding energy positions are characteristic of Pb^2+^, confirming that lead predominantly exists in the +2 oxidation state. The nearly identical peak positions and line shapes for the DMPO4_Pb and Reference_Pb samples indicate that the chemical environment and oxidation state of Pb remain unchanged upon incorporation of the DMPO4 interlayer.

From the UPS spectra (Figure S15b,c in the Supporting Information), the work functions of the films were determined using the relation:

Work function(Φ)=Fermi energy+valence band edge+cutoff
energy

For the reference perovskite film, Φ = 4.07 + 0 + 0.68 = 4.75 eV, whereas the DMPO4-interlayered perovskite exhibits a slightly higher value of Φ = 4.07 + 0 + 0.72 = 4.79 eV, indicating an increase of 0.04 eV upon DMPO4 incorporation. This trend is also observed in Kelvin probe measurements, which yield work functions of 4.78 eV for the reference and 4.90 eV for the DMPO4-modified film, corresponding to a shift of 0.12 eV. In the absence of any detectable shift in the Pb 4f core-level binding energies as revealed by XPS, this work-function increase upon DMPO4 incorporation suggests a subtle modification of the surface potential, likely associated with interfacial dipole formation or improved surface passivation^60^ induced by the phthalocyanine interlayer.

The small increase in *J*_SC_ and, thus, collection efficiency, observed with the incorporation of the interlayer, together with the increased work function revealed by UPS and Kelvin probe measurements, suggest not only a passivation effect from the Zn-phthalocyanine derivative (DMPO4) incorporated at the hole extracting contact but also a more favorable energy alignment at the perovskite/hole extracting contact. Similar effects have also been reported in a work using tin­(II) phthalocyanine in carbon-based flexible perovskite cells published during the preparation of a revised version of our manuscript.^61^

To investigate the impact of the DMPO4 interlayer on perovskite crystallization, we conducted XRD analysis on two device architectures: glass/ITO/DMPO4/­Perovskite and glass/ITO/­Perovskite (reference), as presented in Figure 7. The XRD patterns confirm that both perovskite films exhibit a cubic polycrystalline phase with Miller indices assigned to each reflection according to the literature.^62–64^ While the diffraction peaks appear at the same positions for both samples, differences in full width at half-maximum (fwhm) values indicate variations in crystallinity. A lower fwhm corresponds to higher crystallinity, suggesting improved film quality.^65^ The DMPO4-based perovskite films show significantly narrower peaks with lower fwhm values, particularly along the [100] and [200] planes, with values of 0.142 and 0.169, respectively, compared to 0.166 and 0.183 for the reference sample. These results highlight the enhanced crystalline quality of the DMPO4-modified films.

**Figure 7:**
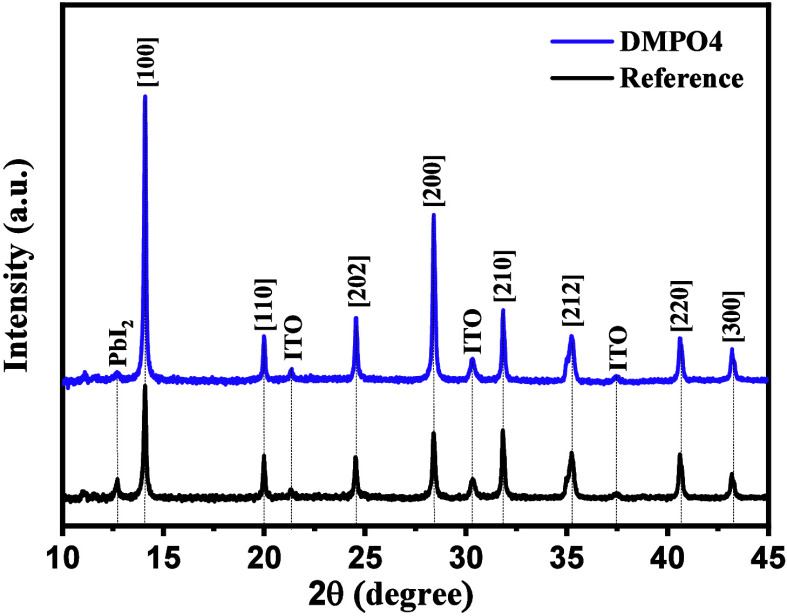
Comparison of thin film XRD patterns between DMPO4-modified perovskite (purple) and reference perovskite (black) films (ITO/MeO-2PACz/­DMPO4/­CsFAMA vs ITO/MeO-2PACz/­CsFAMA).

Additionally, the intensity of the diffraction peaks differs between the two samples, with the DMPO4-based perovskite showing higher peak intensities, particularly along the [100] direction, indicating a preferential crystal orientation that can facilitate charge transport and improve device performance. Although DMPO4 is located at the buried HTL/perovskite interface, it can influence perovskite crystallization during the initial stages of film formation by modifying the interfacial energy landscape and nucleation conditions. This interfacial growth modulation promotes bottom-up crystallization with a reduced heterogeneous nucleation density, leading to enhanced orientation and improved crystalline order. Furthermore, XPS analysis reveals no detectable shift in the Pb 4f core-level binding energies upon DMPO4 incorporation, suggesting that the bulk chemical state of the perovskite remains unchanged. Together with the reduced interfacial defect density revealed by SCLC analysis, these structural improvements are consistent with a growth process governed by interfacial passivation rather than bulk compositional changes. Moreover, the PbI_2_ peak intensity is significantly reduced in the DMPO4-treated film, indicating better purity of the perovskite phase and reduced residual lead iodide. The suppression of PbI_2_ is beneficial because excessive PbI_2_ aggregates inside the perovskite layer can act as recombination centers, negatively affecting the device efficiency and stability. These findings confirm that the incorporation of DMPO4 leads to improved crystallinity, enhanced crystal orientation, and better phase purity, all of which contribute to superior optoelectronic properties and device performance.

Scanning electron microscopy (SEM) was employed to investigate the grain size distribution of the samples. As shown in Figure 8a,b, both films exhibit uniform and compact morphology with grain sizes predominantly in the range of hundreds of nanometers. The grain-size distribution follows a log-normal profile centered around ∼200 nm with sizes spanning approximately 50–450 nm. In the case of the DMPO4-modified film (Figure 8c), the grains are distributed between 150 and 450 nm, whereas the reference film shows a broader distribution extending from 50 to 450 nm (Figure 8d). Notably, a larger fraction of grains (∼22%) is concentrated in the 250–300 nm range for the DMPO4 film, compared to ∼16% of grains in the 200–250 nm range for the reference. This shift toward larger grain sizes is consistent with the enhanced crystallinity and preferential orientation observed in XRD measurements, indicating that the structural improvements induced by the DMPO4 interlayer extend throughout the film and are reflected at the surface, in line with reports for interfacial molecular systems in the literature.^66^ The resulting shift toward larger grains suggests a reduced grain boundary density, which is generally beneficial for suppressing nonradiative recombination and minimizing charge carrier losses.^67^ Furthermore, the improved morphology is expected to suppress shunt pathways and reduce optical scattering losses, ultimately contributing to the enhanced device performance. As a result, the DMPO4-modified film is likely to exhibit superior optoelectronic properties with enhanced charge transport and reduced recombination losses, making it more suitable for high-performance devices.

**Figure 8:**
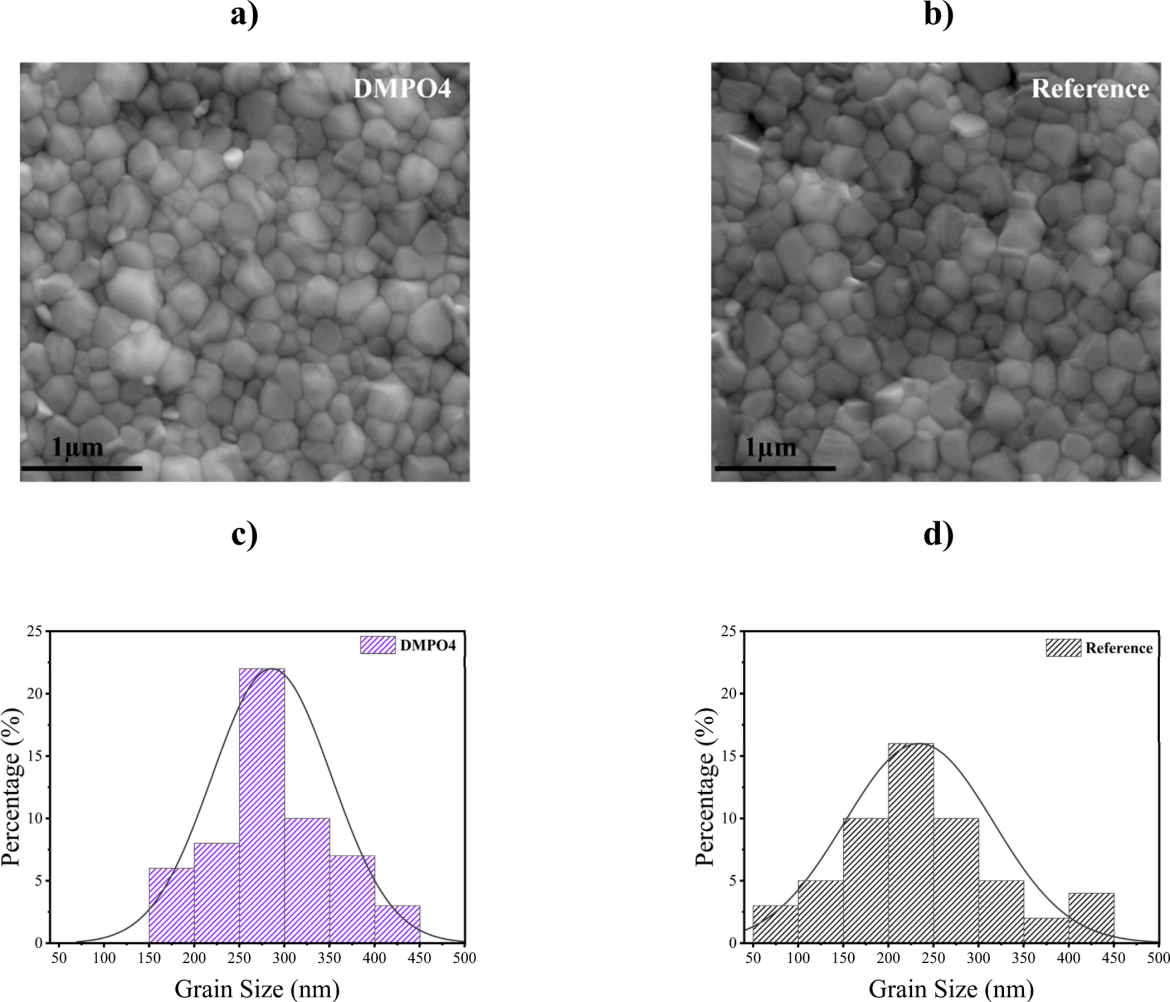
Top-view SEM images of perovskite films on glass ITO: (a) Perovskite morphology with a DMPO4 interlayer (glass/ITO/DMPO4/perovskite) and (b) reference morphology (glass/ITO/perovskite). Grain size distribution histograms derived from SEM analysis are shown in (c) the DMPO4 and (d) reference films. The statistical distributions highlight the crystal grain size evolution before and after the incorporation of the DMPO4 interlayer.

We further investigated the stability of unencapsulated perovskite solar cells (PSCs) under specific conditions. The study followed the ISOS-D-1 protocol, which implies that the cells are stored in the dark in ambient conditions at a controlled temperature of 23 ± 4 °C.^68,69^ Figure 9 shows the time evolution of the PCEs measured at STC. The shelf life stability data reveal that DMPO4 devices are slightly better at preserving their efficiency over time. Although standard deviations are high (but smaller for the devices with the interlayer), the average *T*_80_ for DMPO4 devices was 1347 h, whereas it was 1134 h for the reference devices.

**Figure 9:**
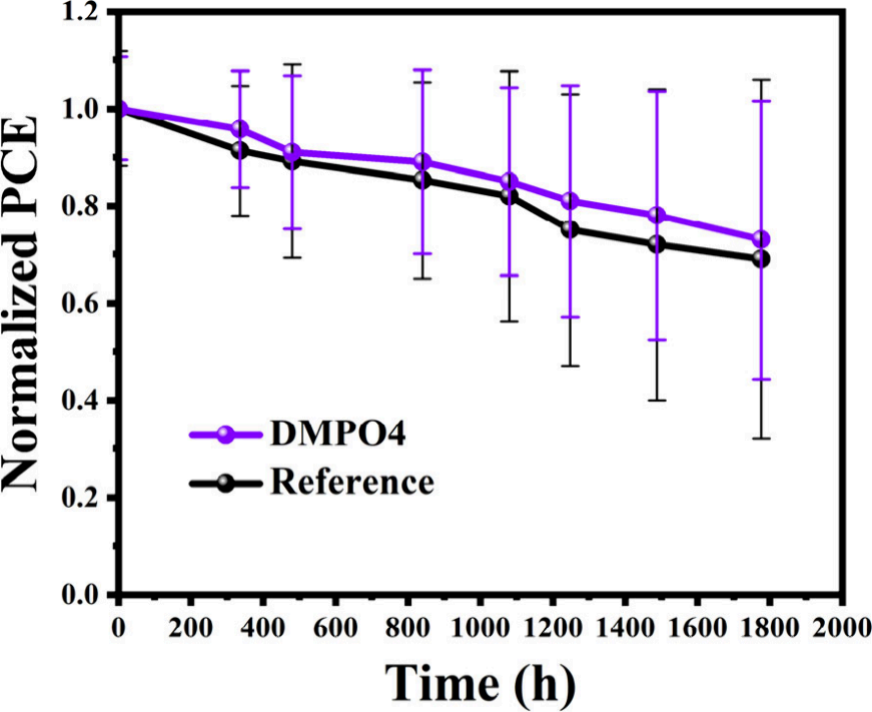
Comparative long-term stability of perovskite devices: The normalized power conversion efficiency (PCE) variation of DMPO4-treated and reference devices was monitored over 1800 h under dark storage conditions (RH < 40%) at room temperature (ISOS-D-1). Stability measurements were conducted periodically under 1 sun illumination (AM1.5G, 100 mW/cm^2^) in an ambient environment.

## 4 Conclusions

In conclusion, the incorporation of the aryloxy-substituted phthalocyanine DMPO4 as an interlayer in perovskite solar cells has yielded advancements in various important aspects of cell performance. The notable increase of about 10% in power conversion efficiency (PCE) in relative terms, rising from 18.4% in reference cells to 20.2% in DMPO4/perovskite cells, is primarily attributed to the higher open-circuit voltage (*V*_OC_). Furthermore, DMPO4-based perovskite devices have extended carrier lifetimes, as demonstrated by longer TPV and TRPL decay times, indicating more effective charge extraction and reduced nonradiative recombination processes. In addition, SCLC measurements reveal a reduced interfacial trap density in DMPO4-based devices, supporting the role of DMPO4 as an interfacial passivation layer. The improved crystallinity, characterized by larger grain sizes and narrower fwhm values in DMPO4/perovskite cells, highlights the role of DMPO4 in enhancing the film quality of the overlying perovskite semiconductor. Moreover, the ability of DMPO4 to lower reverse saturation currents (*J*_0_) as well as the ideality factor reflects its role in reducing recombination and defect densities. Even when measured under indoor lighting conditions, DMPO4/perovskite cells maintain their superior performance, boasting a higher PCE of 30.1% compared to the reference cells of 27.3%. Our ISOS-D-1 stability tests show that the average *T*_80_ for DMPO4 devices was 1347 h, compared to 1134 h for the reference devices, indicating slightly improved stability. Overall, these findings collectively emphasize the significant and positive impact of phthalocyanine molecular interlayers on both the performance and quality of perovskite solar cells, making it a promising avenue for advancing this perovskite technology. Notably, the one-pot synthesis described here is a cost-effective method with a low *E*-factor and may offer a versatile approach for the synthesis of functionalized phthalocyanines for use in organic electronics and optoelectronics.

## Supplementary Material



## Notes

Further synthetic details, *E*-factor calculation, cost analysis, ^1^H and ^13^C NMR spectra, MALDI/TOF spectrum, Fluorescence characterization, and cyclic voltammetry of DMPO4; extended methods for DFT calculations, IPCE spectra, SCLC measurements, and XPS and UPS spectra (PDF)

## †

UOSD Medicina di Precisione in Senologia, Fondazione Policlinico A. Gemelli, L.go A. Gemelli 8, 00168 Roma, Italy
