# Supplementary material for: Improved Perovskite Solar Cells with an Environmentally Friendly Phthalocyanine Hole Extracting Interlayer
Source: ACS Appl Energy Mater. 2026 Feb 26;9(5):2541–54. doi: 10.1021/acsaem.5c03517 (PMC12977231; doi:10.1021/acsaem.5c03517)
Supplement: Supplementary file 1 [file ae5c03517_si_001.pdf]

Supporting Information

Improved Perovskite Solar Cells with an Environmentally Friendly Phthalocyanine Hole Extracting Interlayer

Suresh K. Podapangi,[a,b‡] Laura Mancini,[c‡] †, Daimiota Takhellambam,[a‡] Jie Xu,[a] Luigi Angelo Castriotta,[a] Giuseppe Mattioli,[c] Venanzio Raglione,[b] Federica Palmeri,[c,d] Daniela Caschera,[e] Anatoly P. Sobolev,[f] Antonio Cricenti,[b] David Becerril Rodriguez,[b] Marco Luce,[b] Aldo Di Carlo,[a,b] Gloria Zanotti[c]* and Thomas M. Brown[a]**

[a] S.K. Podapangi, D.Takhellambam, J. Xu, L.A. Castriotta, T.M. Brown, A. Di Carlo, C.H.O.S.E. (Centre for Hybrid and Organic Solar Energy), Department of Electronic Engineering, Tor Vergata University of Rome, 00133, Rome, Italy.

[b] S.K. Podapangi, V. Raglione, A. Cricenti, D. Becerril Rodriguez, M. Luce, A. Di Carlo, Istituto di Struttura della Materia, Consiglio Nazionale delle Ricerche (ISM-CNR), via del Fosso del Cavaliere 100, Rome 00133, Italy.

[c] L. Mancini, G. Mattioli, F. Palmeri, G. Zanotti, Istituto di Struttura della Materia, Consiglio Nazionale delle Ricerche (ISM-CNR), Strada provinciale 35d/9, Montelibretti, Rome 00010, Italy.

[d] F. Palmeri, Department of Chemistry, La Sapienza University of Rome, P. le Aldo Moro 5, 00185 Rome, Italy.

[e] D. Caschera, Istituto per lo Studio dei Materiali Nanostrutturati (ISMN), Consiglio Nazionale delle Ricerche (CNR), Strada provinciale 35d/9, Montelibretti, Rome 00010, Italy.

[f] A. P. Sobolev, Istituto per i Sistemi Biologici (ISB) Consiglio Nazionale delle Ricerche (CNR), Strada provinciale 35d/9, Montelibretti, Rome 00010, Italy.

‡These authors contributed equally.

†Present address: UOSD Medicina di Precisione in Senologia, Fondazione Policlinico A. Gemelli, L.go A. Gemelli 8, 00168, Roma

*Corresponding Authors:

thomas.brown@uniroma2.it, gloria.zanotti@ism.cnr.it, aldo.dicarlo@uniroma2.it

Table S1: Variation of the experimental conditions for the synthetic procedure. Amount of 4-nitrophthalonitrile: 500 mg (2.89 mmol). Solvent: DMF (4 mL). For sake of reproducibility, each reaction has been performed twice.

Phthalonitrile/phenol molar ratio	Base	S_NAr step temperature	DBU in the second step	Yield (%)
1.00:1.10	DBU (2.00 equiv)	60	Yes	44
1.00:1.10	DBU (2.00 equiv)	60	No	29
1.00:1.10	K_2CO_3 (3.00 equiv)	60	Yes	29
1.00:1.02	DBU (3.00 equiv)	25	Yes	41

General procedure:

In a two-necked 25 mL flask equipped with a reflux condenser, 3,5-dimethylphenol and the chosen base were added and stirred in 4 mL of dimethylformamide for 20 min at room temperature. Then, 0.500 g (2.89 mmol) of 4-nitrophthalonitrile was added, and the mixture was allowed to react at the selected temperature until complete consumption of the reagents, monitored by TLC chromatography. Subsequently, zinc acetate dihydrate and, optionally, a second portion of DBU were added to the mixture, which was then stirred at 150°C for 20 h. The reaction was then cooled, treated with 3.5 mL of HCl 1.0 M, filtered, and washed with 5 mL of water. The resulting crude product was purified by filtration on a silica pad (6 g) using 60 mL of a 5:1 (v/v) petroleum ether/tetrahydrofuran mixture. The solid obtained was further purified by Soxhlet extraction using methanol as the solvent.

General equation for the calculation of E-factor:

$$E\text{-}factor = \frac{Mass\ of\ total\ waste\ (g)}{Mass\ of\ product\ (g)}$$

E-factor calculation for the best-yielding reaction (including **water** and **water-based solutions**):

$$\frac{0.388+0.880+0.500+3.79+0.171+0.440+\mathbf{3.500}+\mathbf{5.000}+6.000+31.114+14.240-0.335}{0.335} = 196.1$$

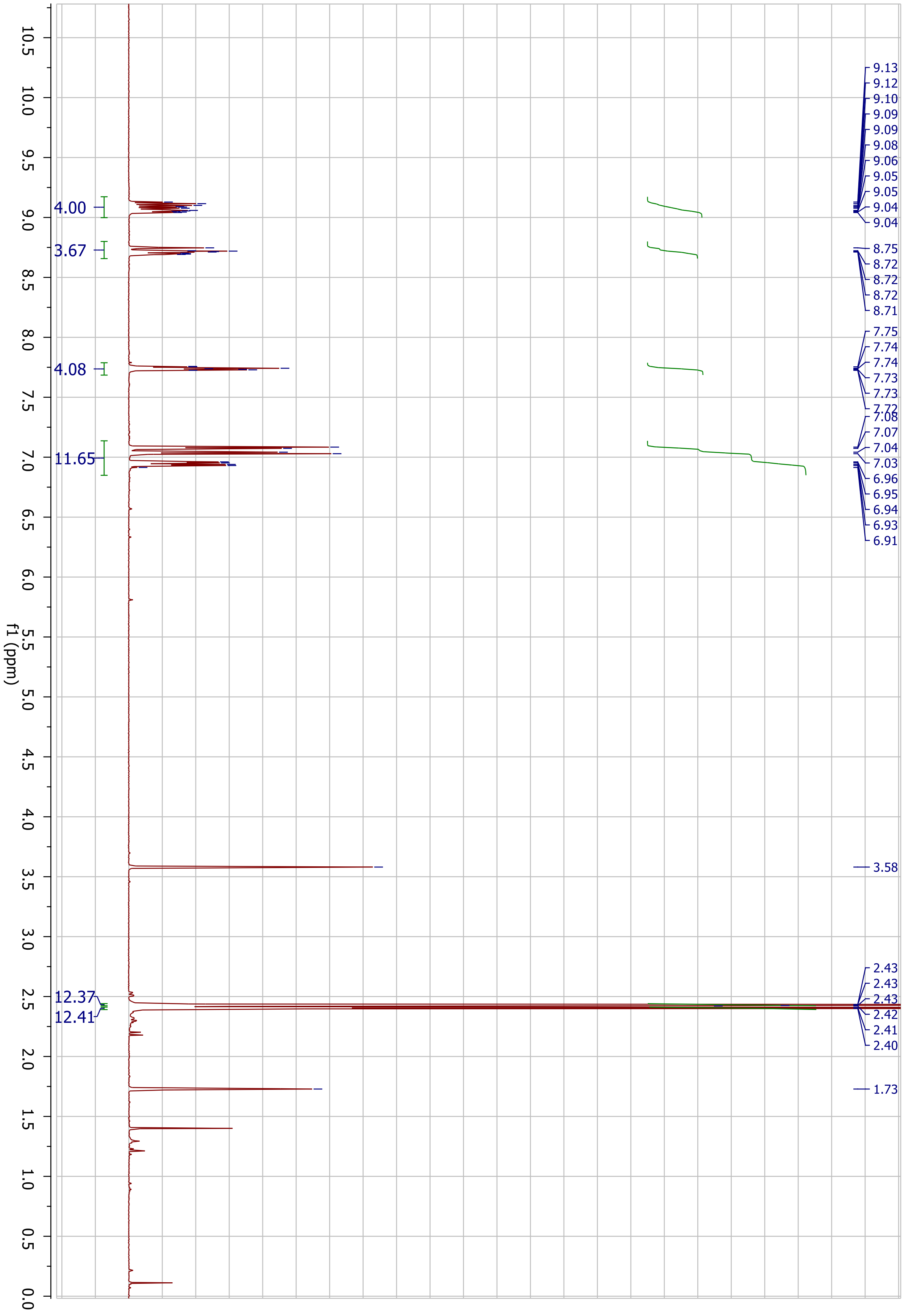

Figure S1. ^{1}H NMR of DMPO4 in THF-d$_8$.

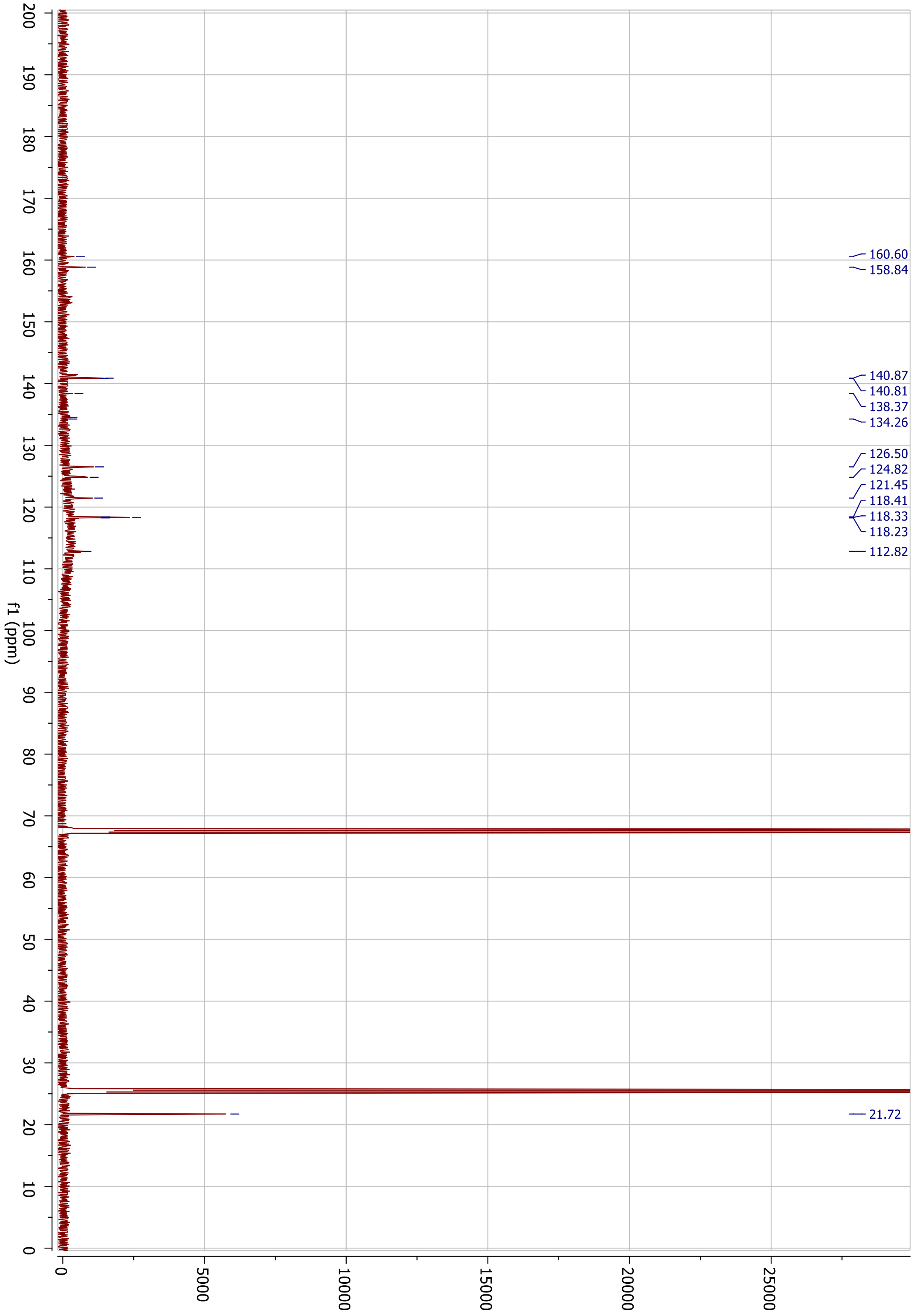

Figure S2. ^{13}C NMR spectrum of DMPO4 in THF-d_8.

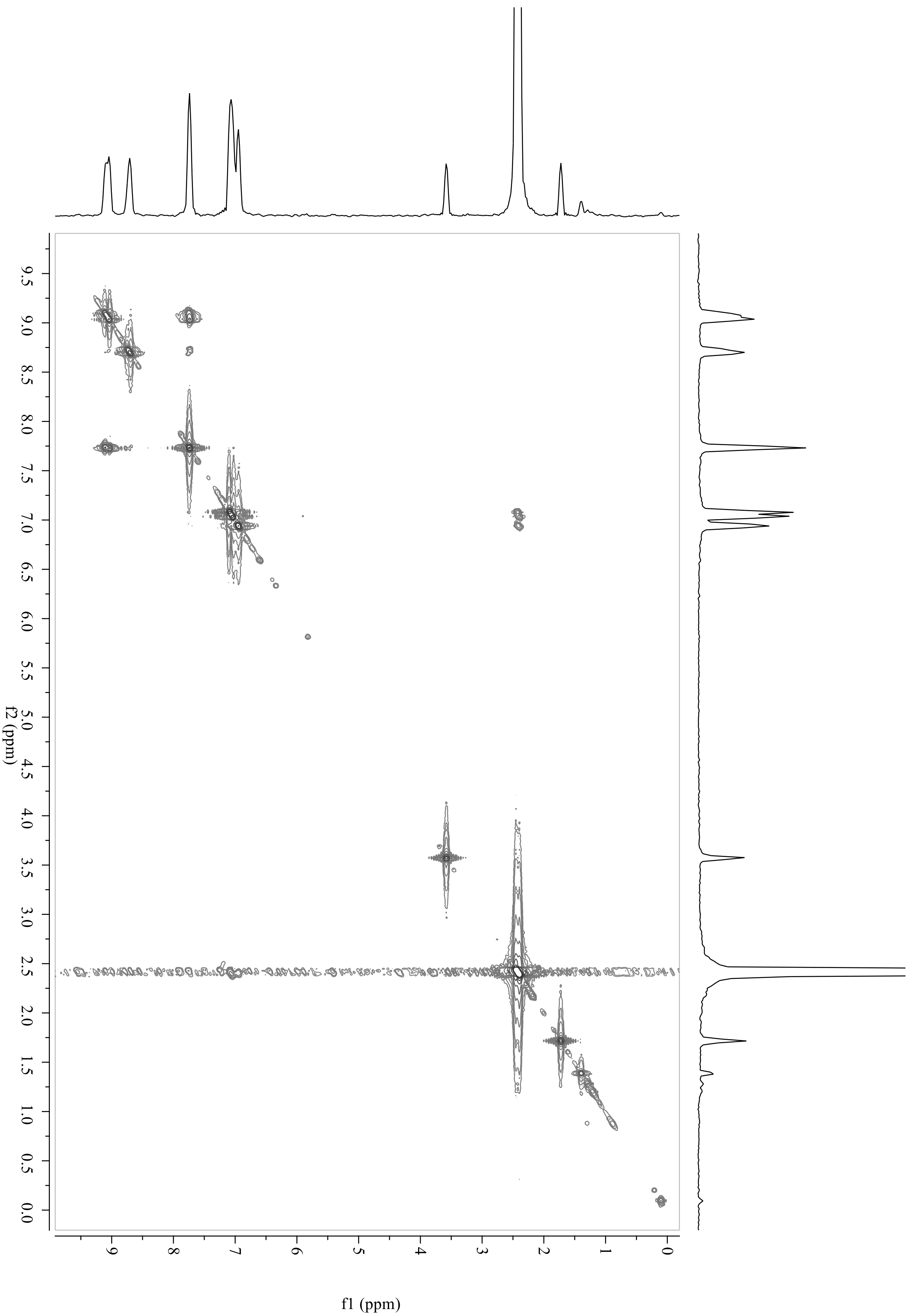

Figure S3. Correlation spectroscopy (COSY) spectrum of DMPO4 in THF-d_8.

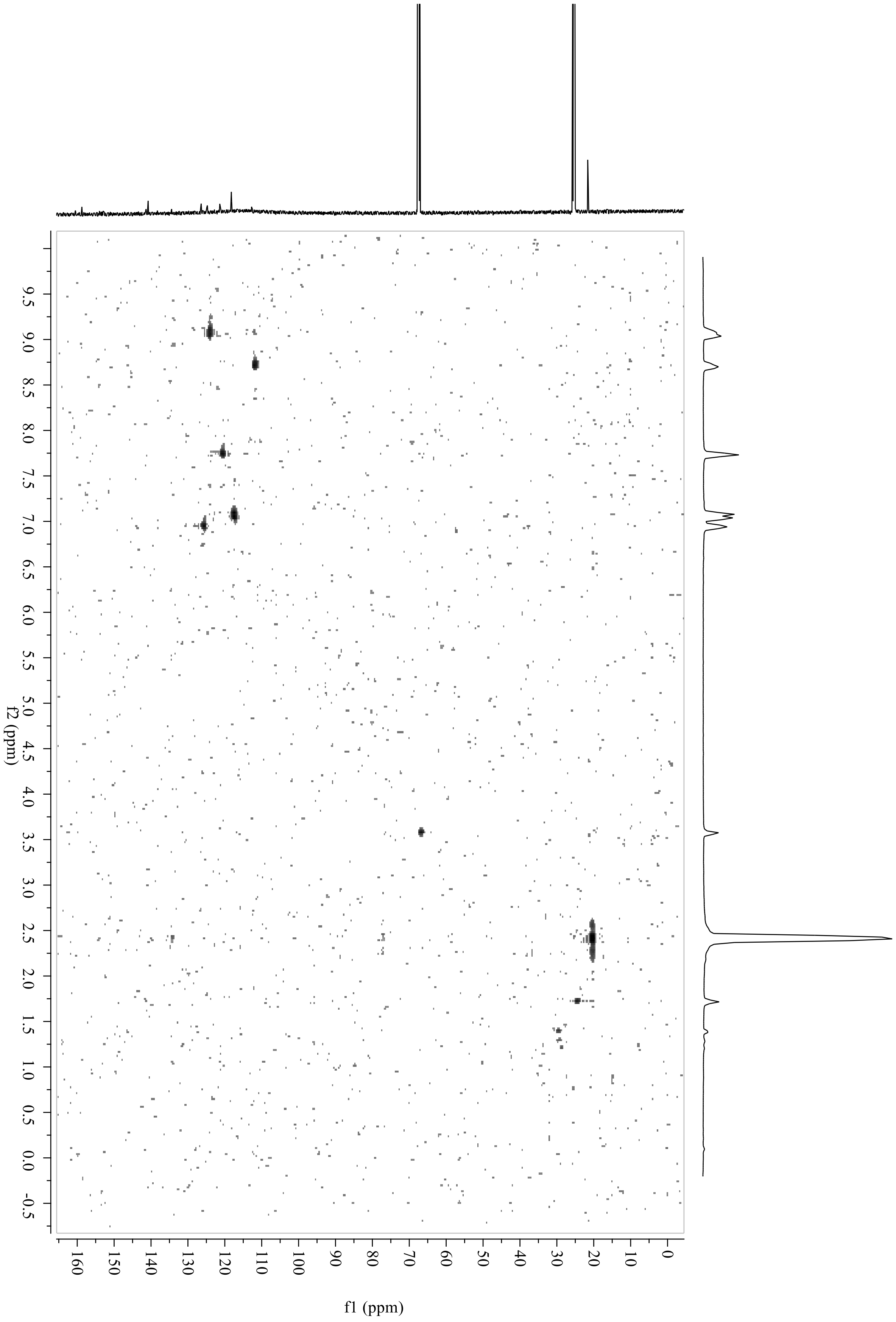

Figure S4. Heteronuclear Single Quantum Coherence (HSQC) spectrum of DMPO4 proton-carbon single bond correlations in THF-d_8.

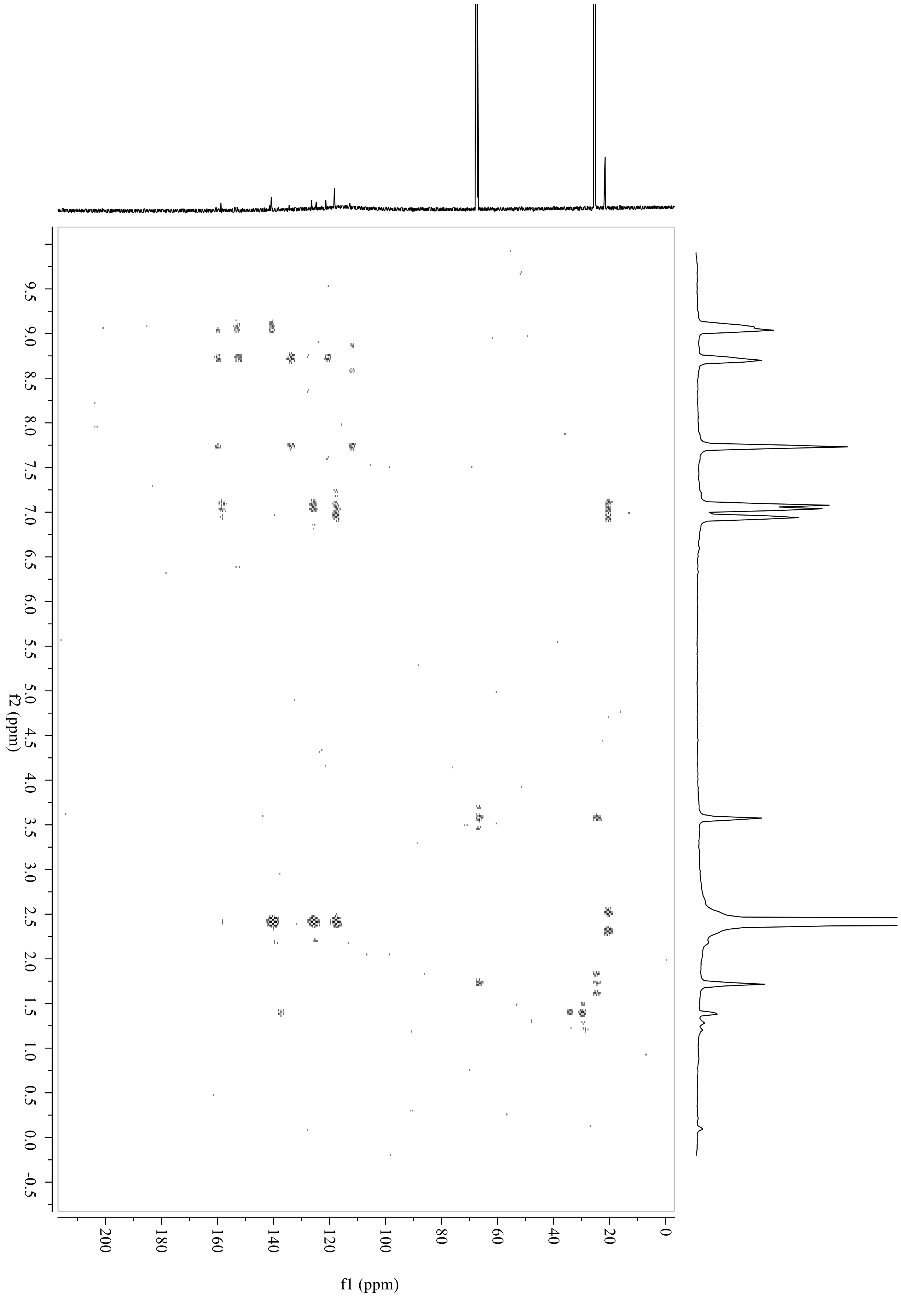

Figure S5. Heteronuclear Multiple Bond Correlation (HMBC) spectrum of DMPO4 in THF-d_8.

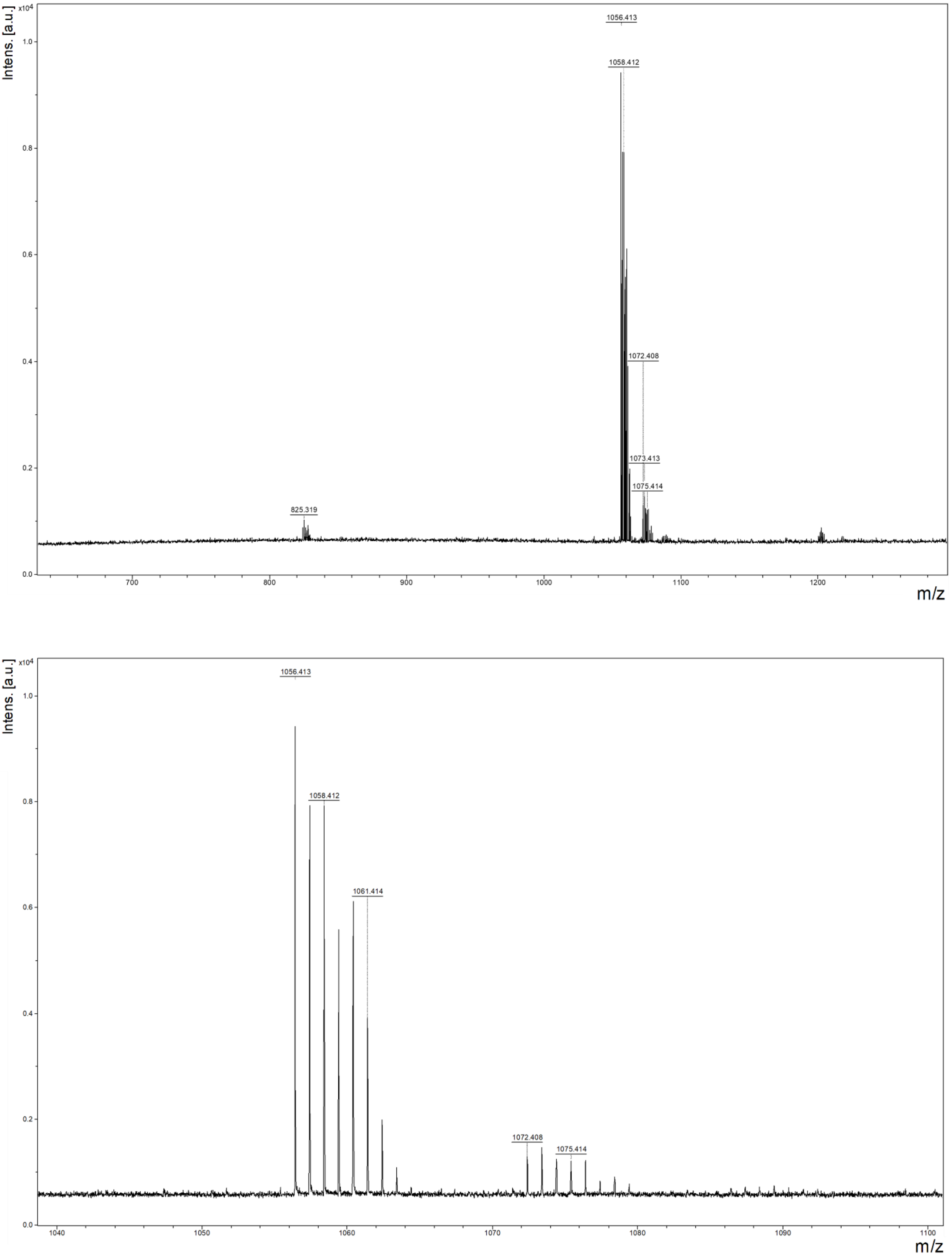

Figure S6. Upper panel: MALDI-TOF spectrum of DMPO4. Lower panel: magnification of the isotopic cluster of the molecular ion of DMPO4.

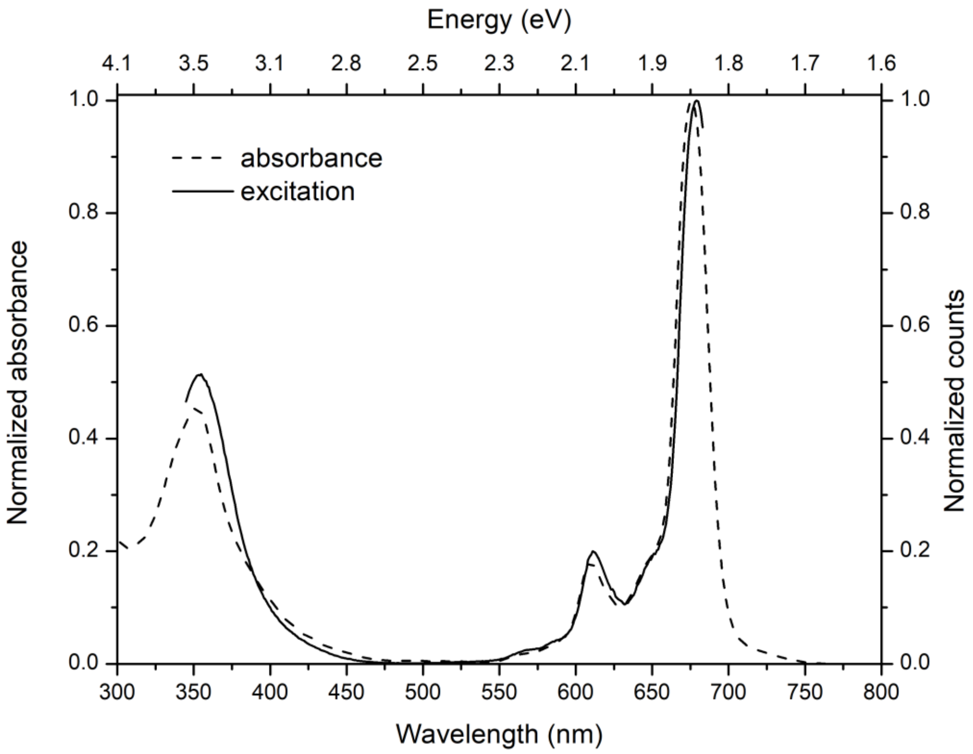

Figure S7. Normalized fluorescence excitation spectrum of DMPO4 (solid curve) in dichloromethane (λ_{em} = 685 nm). The normalized absorption spectrum (dashed curve) in the same solvent is plotted for comparison.

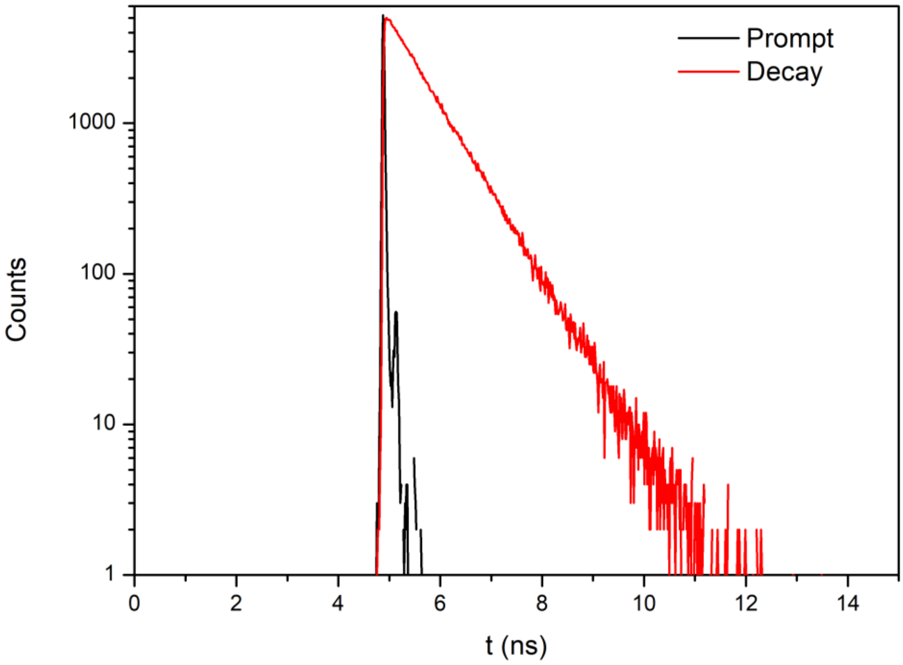

Figure S8. Fluorescence decay of DMPO4 (red curve) in dichloromethane.

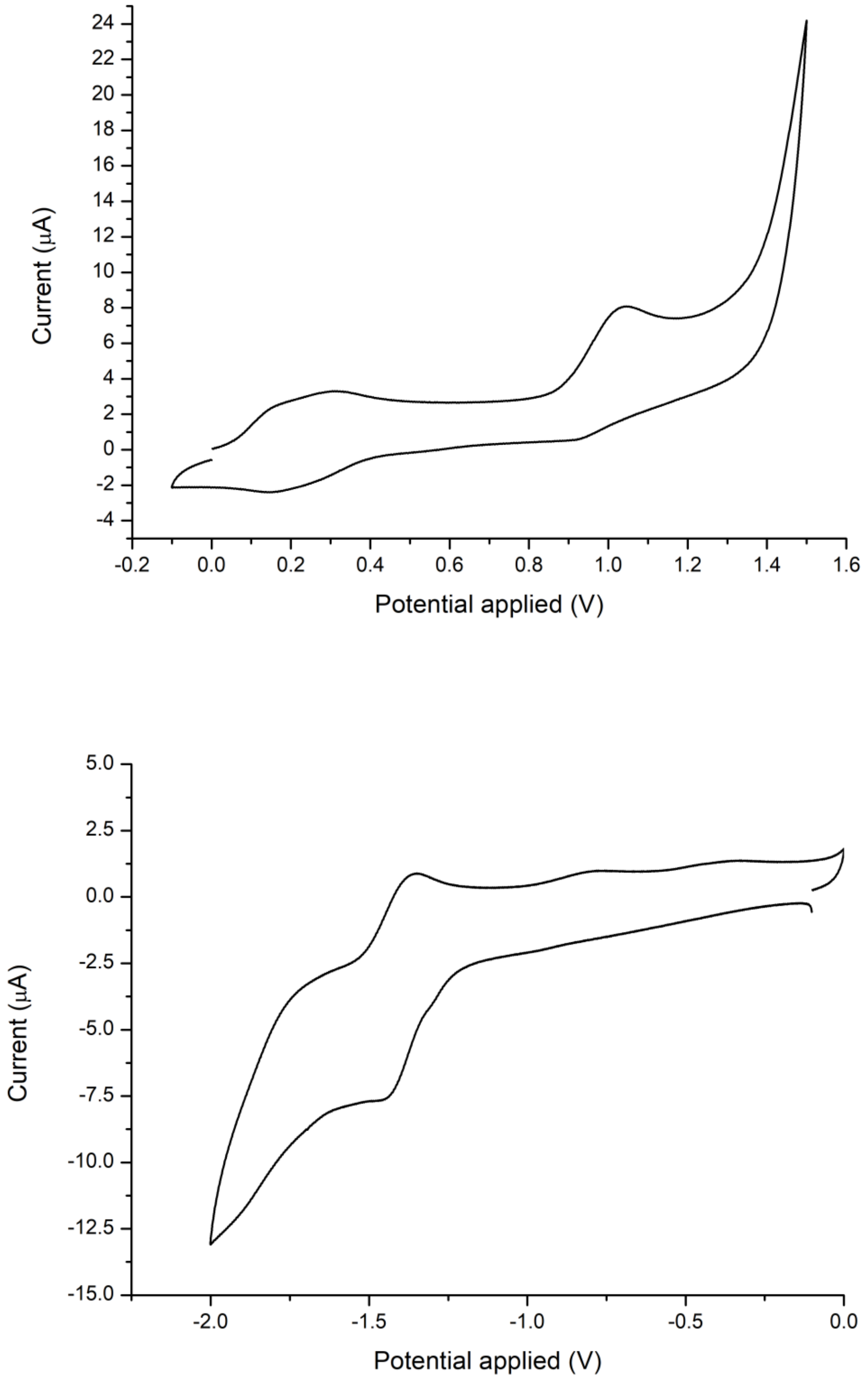

Figure S9. Anodic (upper panel) and cathodic (lower panel) cyclic voltammograms of DMPO4 recorded in freshly distilled dichloromethane containing 0.1 M tetrabutylammonium tetrafluoroborate as supporting electrolyte.

Extended Computational Methods. The simulations of DMPO4 and ZnTTB have been performed using a multi-level protocol involving different theoretical methods. Particular care has been devoted to selecting theoretical methods able to reproduce the results of electrochemical and optical measurements. The most stable conformers of the molecules have been found using a conformer-rotamer search algorithm (CREST) based on the GFN2-xTB tight-binding Hamiltonian[1–3]. Such structures have been then fully optimized using the ORCA suite of programs[4–6] in a localized-basis-set framework. In detail, the B3LYP hybrid functional[7] and the D3BJ dispersion correction[8] have been used. The Kohn-Sham orbitals have been expanded on an all-electron def2-TZVPP Gaussian type basis set[9,10]. The corresponding def2/J basis has been also used as an auxiliary basis set for Coulomb fitting in a resolution-of-identity/chain-of-spheres (RIJCOSX) approximation. The obtained structures have been then used as starting points for a series of calculations using a representative set of exchange-correlation functionals, as reported in detail in Table S2 (page S-15). This set includes the revised PBE0[11] and the M06-2X[12] global hybrid functionals, as well as the CAM-B3LYP[13], the ωB97X[14] and the ωr^2SCAN-X[15] range-separated hybrid functionals, using the same basis set employed for B3LYP calculations. This same set of functionals with the same basis sets has been also used for the calculations of absorption spectra in the sTDDFT framework[16], whose results are reported in Table S2.

Electrochemical potentials E0/+ and E0/- of the molecules have been calculated as discussed in detail elsewhere[17] using, again, the same basis set discussed above. The corresponding values are reported in Table S2. In all calculations the molecules have been surrounded by an implicit dichloromethane solvent using the conductor-like polarizable continuum model (CPCM)[18] and fully optimized, in order to calculate at DFT level electronic and solvation energies. Thermochemical properties of the molecules have been calculated using both the GFN2-xTB and B3LYP levels of theory introduced above. The corresponding redox potentials have been then calculated as ΔG values between neutral and charged species. We remark that our protocol provides best results when using ωB97X, particularly suited for the calculation of absorption spectra of large molecules with sTDDFT due to its large fraction of exact-exchange[16], and M06-2X, particularly suited for the calculation of electrochemical properties of organic molecules[19]. The optimized structure of DMPO4 in Cartesian coordinates is reported below.

DMPO4

Coordinates from ORCA-job dmpo4-b3lyp-cpcm-def2tzvpp

Zn	-0.00142944413862	0.00019708882230	-0.10374614652127
N	1.80019497367653	-0.86510566324281	-0.05522698600696

C	2.98912492203602	-0.19263248393155	-0.02465106408719
N	3.17776574608199	1.11937894163399	-0.02473572581508
C	2.21269977021875	2.02907226782558	-0.05327647463020
N	0.86331638648725	1.80301441424649	-0.08816398634473
C	0.19148295775317	2.99264532750111	-0.10793127910649
N	-1.12007095438970	3.18127173856525	-0.14269055646820
C	-2.02932634072729	2.21557584855971	-0.16340190214399
N	-1.80311207010655	0.86569543257059	-0.15235744131240
C	-2.99214525626092	0.19327914676251	-0.18057331171289
N	-3.18067779336895	-1.11871206741205	-0.18156689001560
C	-2.21550918006985	-2.02837058115846	-0.15390946516493
N	-0.86617490675151	-1.80253397753052	-0.11755003991264
C	-0.19446802092356	-2.99229041175124	-0.09758959750514
N	1.11707970203930	-3.18078405526991	-0.06153885622112
C	2.02632941871888	-2.21499961602691	-0.04102060164270
C	3.46351218578594	-2.44087672184466	0.00160113999827
C	4.24407597853498	-3.59415896721183	0.03127666234742
C	5.62049945649760	-3.45538256001867	0.07156689182568
C	6.21677180820315	-2.18148248144682	0.08187044699042
C	5.45118847552796	-1.01994692483933	0.05144506378645
C	4.07133217860452	-1.17247847268026	0.01184938064835
H	5.90542945786813	-0.04137218317888	0.05863557184342
O	7.58549350253383	-2.17564038041287	0.12038615728628
C	8.24687210489286	-0.94914674758791	0.16045632160805
C	8.67298195700782	-0.37900676296715	-1.02504239281558
C	9.37945735965747	0.82612237852243	-0.99157996670384
C	9.62948331492678	1.41924495310883	0.24403930830834
C	9.19714547412025	0.84432030732480	1.44219300314914
C	8.49905889024231	-0.36007214168460	1.39067884062845
H	8.14917736801995	-0.84103529703652	2.29447004215127
C	9.45534983882745	1.52615937401381	2.75865832261086
H	9.46125308803225	0.81094362809129	3.58055201062481
H	8.67630667220332	2.26356272570548	2.96885551192165
H	10.40907129777532	2.05388009893247	2.74950696924607
H	10.17865440225637	2.35259183925323	0.27746047757411
C	9.85989166245553	1.45752322221840	-2.27034761344733
H	10.34812841402556	2.41199521294351	-2.07947191594635
H	10.57195970189450	0.80726174799637	-2.78284313045303
H	9.02901140993573	1.62927506607353	-2.95725371771226
H	8.45537947107527	-0.87221342026119	-1.96328622887889
H	6.26557999072720	-4.32266185389256	0.09634293108387
H	3.79020053781644	-4.57537575761419	0.02414492743196
C	-1.17479187553057	-4.07483443623638	-0.12278991066500
C	-1.02428414552401	-5.45571895279308	-0.11790284634660
C	-2.18674788349699	-6.21993792796623	-0.14970285531450
C	-3.45935309821526	-5.62210009635372	-0.18607658296867
C	-3.59621072349968	-4.24506446782670	-0.19081839513003
C	-2.44220697653490	-3.46580062035524	-0.15842288002804
H	-4.57627074158720	-3.78957019237173	-0.21899046501505
H	-4.32700385942608	-6.26671266975794	-0.21073870942416
O	-2.18391642100603	-7.58915374254272	-0.15376719362980

C	-0.96291268226764	-8.25698370422205	-0.07835089551853
C	-0.42678917829136	-8.54128923443308	1.16950415943462
C	0.77137091215833	-9.24586792111067	1.25481996470976
C	1.40047636904885	-9.64302211694213	0.07131720910939
C	0.86104060286999	-9.36085597387444	-1.18137158528639
C	-0.34258666129119	-8.65337600285774	-1.24843800648489
H	-0.79119664221275	-8.40552581656125	-2.20145929480775
C	1.54709619905380	-9.80866344038608	-2.44369246723235
H	0.95804567568142	-10.57310201901559	-2.95546676640082
H	1.66975179799001	-8.97718188356944	-3.13973849486136
H	2.52990399436153	-10.22681452914151	-2.23111356206856
H	2.33621687020568	-10.18610281995834	0.13051884871324
C	1.36724780002894	-9.59248724419679	2.59256504211934
H	1.16194610596858	-10.63510281142938	2.84795794400057
H	2.45063069420724	-9.46867257213942	2.58437044326994
H	0.95315957913444	-8.96899075588826	3.38397973484229
H	-0.94221931735277	-8.20782535844843	2.06032560314562
H	-0.04678459676572	-5.91171022623858	-0.09098507830515
C	-4.07445701205186	1.17327337281904	-0.21242709159948
C	-5.45444460038179	1.02086144946550	-0.24820026123928
C	-6.21992477729684	2.18251929878620	-0.27524062897199
C	-5.62359548239638	3.45634171305483	-0.26489456797651
C	-4.24707274474920	3.59502158844743	-0.22773948763694
C	-3.46653998021640	2.44163423578386	-0.20187796832263
H	-3.79312969719691	4.57620568888878	-0.22020692616946
H	-6.26869706760091	4.32367595104469	-0.28678939944626
O	-7.58894111751928	2.17683039134345	-0.30511088053088
C	-8.25047589199457	0.95213312073890	-0.37692991322261
C	-8.71673149527462	0.37638480205541	0.79042359910585
C	-9.42467085475242	-0.82687416758932	0.72694342503681
C	-9.63595230009884	-1.41199835018250	-0.51948357173225
C	-9.16304855086615	-0.83122895016388	-1.69958715972281
C	-8.46401007620637	0.37072019572142	-1.61835296236016
H	-8.08422718085087	0.85599911244110	-2.50766521152199
C	-9.38107867937600	-1.50422321504706	-3.02781611103300
H	-10.33742339637498	-2.02663870205626	-3.05328722978659
H	-9.35584903785740	-0.78444655767191	-3.84534550897083
H	-8.59947707851462	-2.24479239182729	-3.21624401448909
H	-10.18675983957929	-2.34322491315416	-0.57651108441272
C	-9.94273935881562	-1.46714019255247	1.98645220809534
H	-10.62349149902625	-0.79645657753373	2.51444224542896
H	-10.47556254974956	-2.39146474637269	1.76843956787380
H	-9.12403285773153	-1.69864890956835	2.67082597443610
H	-8.52920775381383	0.86380970182726	1.73810535839172
H	-5.90864031733863	0.04223552994320	-0.25449125890762
C	1.17158760658320	4.07521541270876	-0.08372338384576
C	1.02025615608096	5.45590249201723	-0.08730883209683
C	2.18220033198826	6.22089825467964	-0.05605727000196
C	3.45520925490899	5.62368205086144	-0.02338043582456
C	3.59284286969705	4.24665941312646	-0.02002223287227
C	2.43922270877031	3.46664726149343	-0.05000633657187

H	4.57323803457304	3.79174578799622	0.00614809556372
H	4.32261701172079	6.26866169129122	0.00037336217064
O	2.17816131797812	7.59022367949501	-0.05541167932713
C	0.95286188804797	8.25481228237845	-0.06578367292800
C	0.39156354145991	8.60994029788240	-1.28032439897675
C	-0.81749987059467	9.30713543605877	-1.29864905706693
C	-1.42601160169772	9.62175558679845	-0.08368335830274
C	-0.85746897863534	9.26859700182787	1.14136120000275
C	0.35070465113050	8.57206686592953	1.14102424449812
H	0.81921735332993	8.26789712422303	2.06766080080863
C	-1.51985146500562	9.64852218168904	2.43807939851419
H	-2.59746754095579	9.75256641761672	2.31469315258794
H	-1.13596490613196	10.60544728269375	2.80109900817900
H	-1.32863458067372	8.90540898304646	3.21214914242568
H	-2.36979071262778	10.15386207089483	-0.09094674092630
C	-1.43646034628943	9.72287655879872	-2.60576039472112
H	-1.31483503857911	8.94888346340553	-3.36403001422324
H	-2.49943814129367	9.93110707901355	-2.49002717940368
H	-0.95986445898655	10.62945907480286	-2.98723859695407
H	0.89059958504402	8.33489093116683	-2.20013066809254
H	0.04237908915943	5.91106042080838	-0.11276164718344

Table S2. Energy of frontier orbitals, Electrochemical potentials in dichloromethane, and maxima of the absorption band in the visible-near IR of the molecules DMPO4 and ZnTTB are reported, together with the level of theory used to perform the calculations. Values in parentheses differ for the use of the GFN2-xTB Hamiltonian instead of the B3LYP+D3 functional for the calculation of thermochemical properties. Experimental (exp) values were derived from cyclic voltammetry and optical absorption measurements, as described in the main text.

	**Frontier Orbitals (eV)**			**Redox Potentials (V)**		**Absorption (nm)**
**DMPO4**	**HOMO**	**LUMO**	**Eg**	**E0/+**	**E0/-**	**Q Band**
B3LYP	-4.85	-2.72	2.13	-4.78	-3.24	692
revPBE0	-5.19	-2.86	2.33	-4.79	-3.27	716
M06-2X	-5.90	-2.53	3.37	-5.15 (-5.21)	-3.45 (-3.42)	890
CAM-B3LYP	-5.87	-2.22	3.65	-4.73	-3.41	733
ωB97X	-6.44	-1.66	4.78	-4.83	-3.73	684
ωr^2SCAN-X	-6.65	-1.88	4.77	-4.80	-3.77	[c]
exp	-	-	1.59[a]<br>1.82[b]	-5.28	-3.69	675
**ZnTTB**	**HOMO**	**LUMO**	**Eg**	**E0/+**	**E0/-**	**Q Band**
B3LYP	-4.95	-2.78	2.17	-4.92	-3.19	670
revPBE0	-5.21	-2.86	2.35	-4.92	-3.22	694
M06-2X	-5.90	-2.52	3.38	-5.16 (-5.15)	-3.45 (-3.40)	869
CAM-B3LYP	-5.87	-2.21	3.66	-4.84	-3.31	724
ωB97X	-6.43	-1.64	4.79	-4.93	-3.58	673
ωr^2SCAN-X	-6.65	-1.87	4.78	-4.91	-3.66	[c]
exp	-	-	-	-5.13	-	676

[a]Voltammetric; [b]Optical; [c]ωr^2SCAN-X is not compatible with sTDDFT, yet.

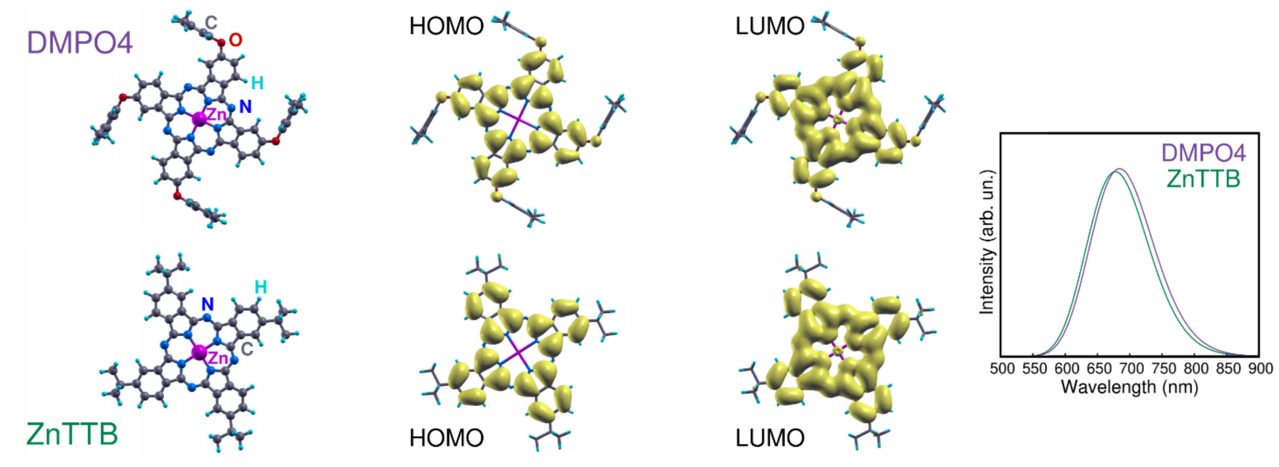

Figure S10. Optimized geometries, $|\psi|^2$ of frontier orbitals and TDDFT absorption spectra in the visible-near IR region of DMPO4 and ZnTTB.

Cost analysis

For the sake of simplicity, Merck has been chosen as the only supplier for reagents, solvents, and purification materials (https://www.sigmaaldrich.com/IT/it) except for phthalonitrile that has been quoted from TCI chemicals (https://www.tcichemicals.com/BE/en/). In all cases, the bulkiest batches available on the online catalogs at a sufficient degree of purity have been chosen and the product number of each chemical has been reported in **Table S2** for sake of clarity. The cost of our synthesis has been estimated by calculating the required amounts of chemicals to produce 1.000 gram of DMPO4 in 44% yield (see the synthesis flowchart in **Figure S11**) and multiplying them by their current prices, rounded to the nearest cent.
Prices have been checked on March 31st, 2025.
The density (d) of HCl 1.0 M has been approximated to 1.00 g/cm^3.

Table S3. Overview of the quoted materials, with densities provided for those available in volumetric units.

Reagent	Supplier	Quoted price (EUR)	Size	EUR/g	EUR/used amount (detailed in **Figure S11**)
3,5 dimethylphenol 144134-100G	Merck	44.00	100.0 g	0.44	0.51
4-nitrophthalonitrile N0524	TCI chemicals	135.00	100.0 g	1.35	2.01
$Zn(OAc)_2 \cdot 2H_2O$ 383058-2.5KG	Merck	507.00	2.5 kg	0.20	0.10
DBU 8032829050<br>d= 1.02 g/cm^3	Merck	3030.00	50.0 kg	0.06	0.24
DMF 8222756190<br>d= 0.948 g/cm^3	Merck	5400.00	190.0 L<br>180.12 kg	0.03	0.34
HCl 1.0 M 1090579025	Merck	261.00	25.0 L<br>25.0 kg	0.01	0.11
Silica gel 60741-25KG	Merck	1740.00	25.0 kg	0.07	1.25
Petroleum ether 50-701009106025<br>d= 0.662 g/cm^3	Merck	707.00	25.0 L<br>16.5 kg	0.04	3.97
THF 360589-20L<br>d= 0.890 g/cm^3	Merck	887.00	20.0 L<br>17.8 kg	0.05	2.12

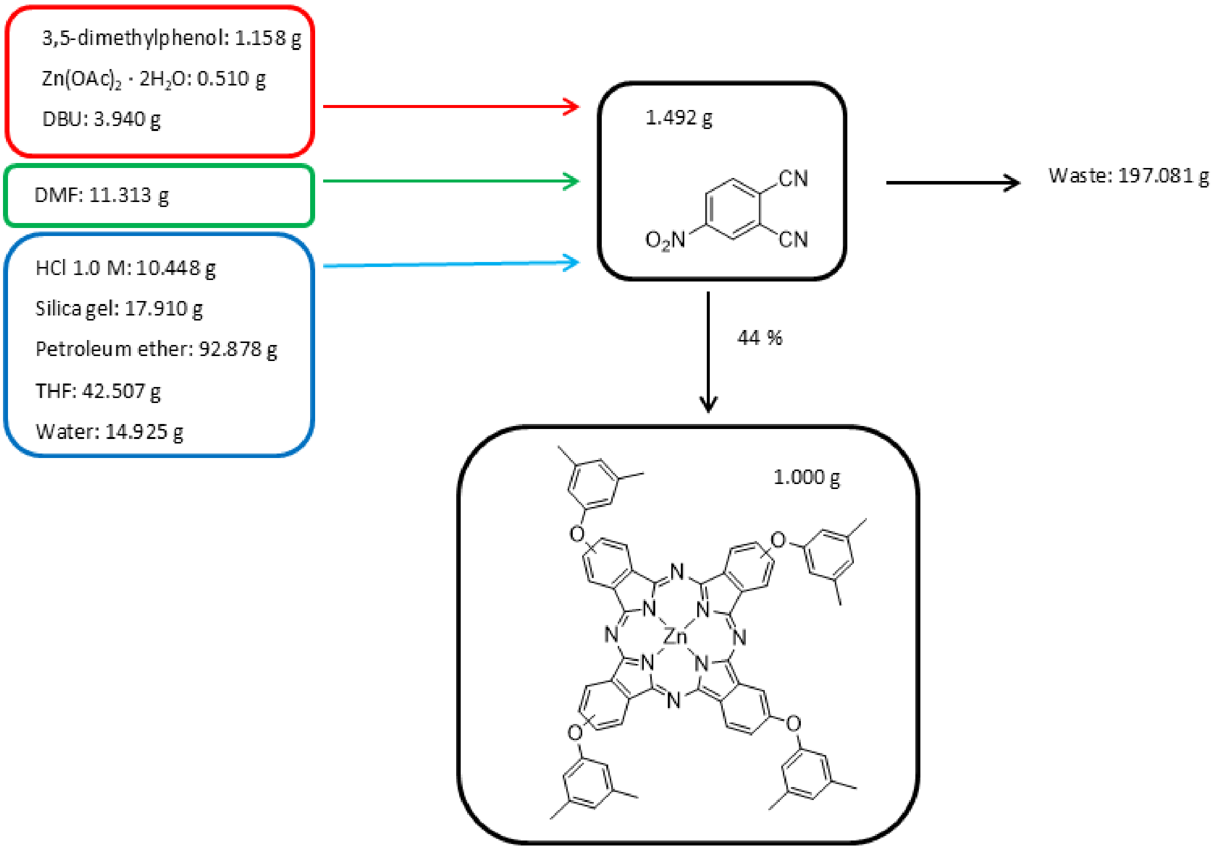

Figure S11. Flowcharts for the synthesis of 1.000 g of DMPO4. Reagents are highlighted in red, solvents are highlighted in green and workup/purification materials are highlighted in blue.

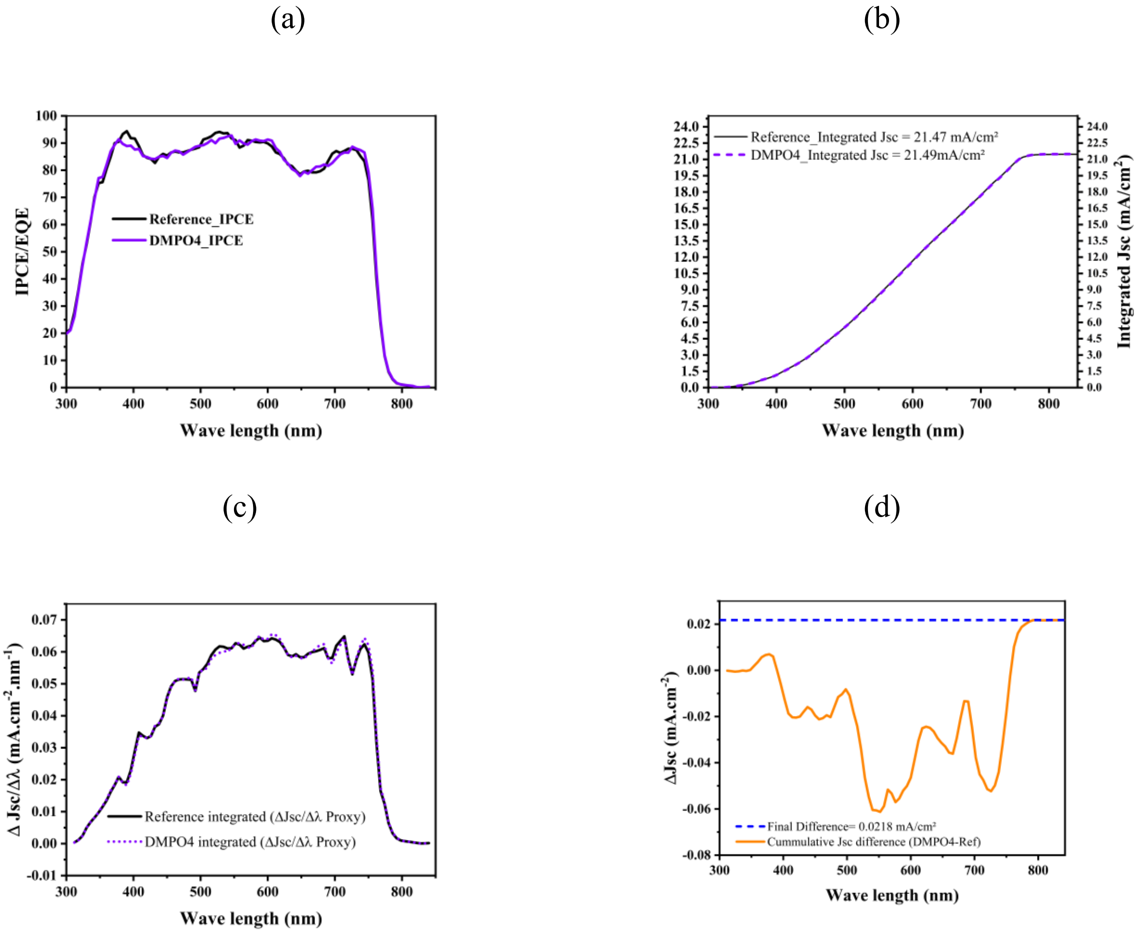

Figure S12. Comparison of the external quantum efficiency (EQE/IPCE) spectra and corresponding photocurrent analyses for the reference (Black line) and DMPO4 (Violet line)-based interlayer devices. *(*a) EQE (IPCE) spectra showing wavelength-dependent photoresponse of both devices, (b) Differential spectral contribution (dJsc/dλ) illustrating the wavelength-resolved current generation under AM1.5 illumination, (c) Cumulative integrated short-circuit current density (Jsc) obtained by integrating EQE(λ) × photon flux(λ) across the solar spectrum, showing nearly identical final Jsc values (Reference: 21.47 mA cm^{-2}; DMPO4: 21.49 mA cm^{-2}), (d) Spectral and cumulative differences (ΔJsc) between the two devices, indicating that local EQE variations are compensated across different wavelength regions, resulting in negligible net difference in total photocurrent.

Although the IPCE spectra of the reference and DMPO4 devices show local differences in spectral shape most notably around ~350–400 nm, ~500–600 nm, and ~700 nm—the spectral contributions to the short-circuit current (i.e., EQE(λ) × photon flux(λ)) offset each other when integrated over the solar spectrum (Figure S12a–b). The cumulative integrated Jsc curves (Figure S12c) clearly show that positive deviations at some wavelengths are compensated by negative deviations at others,

yielding nearly identical final integrated Jsc values (Reference: 21.46818 mA cm^{-2}; DMPO4: 21.48998 mA cm^{-2}; Δ = 0.0218 mA cm^{-2}). The difference plot (Figure S12d) further confirms this spectral cancellation, where localized gains and losses average out across the full wavelength range. We include the EQE(λ) spectra, the corresponding spectral current contributions (EQE × AM1.5 proxy), and the cumulative integrals in the Supporting Information to demonstrate this effect. The remaining difference is well within the experimental uncertainty of the EQE integration.

The integrated short-circuit current density (J_{SC}) calculated from the EQE spectra was found to be consistent within the experimental error for both the Reference and DMPO4 devices. This indicates that the absorbed photons effectively contribute to the photocurrent generation in both cases.

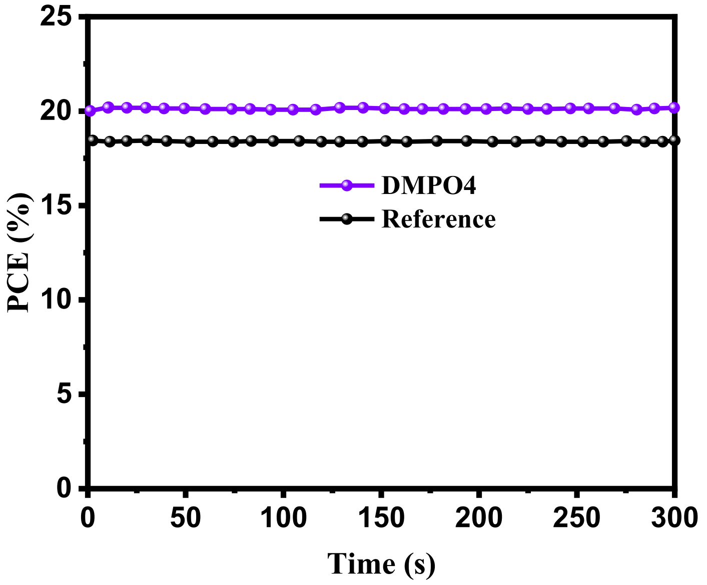

Figure S13. PCE of Reference (black line) and DMPO4 (violet line) best performing devices measured under a bias near the maximum power point.

To further test the interlayer stability and performance, the PCE was measured near the maximum power points of the corresponding devices. The steady-state measurements resulted in a power conversion efficiency (PCE) of 18.44% for the reference device and 20.11% for the DMPO4 device, closely matching the measured values.

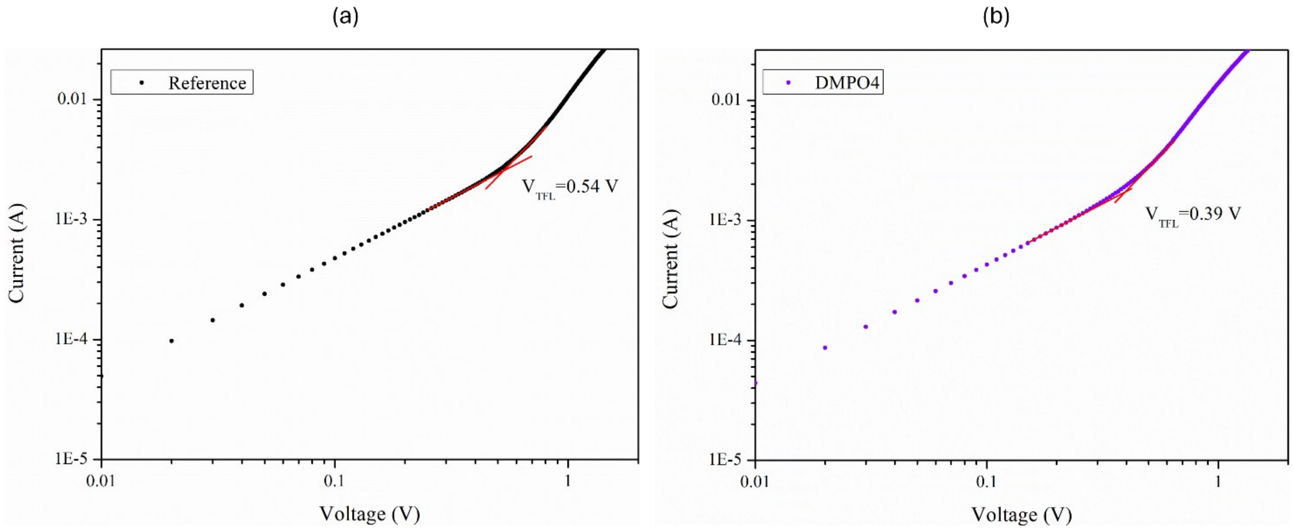

Figure S14: Space-charge-limited current (SCLC) dark I–V characteristics of hole-only devices with the structure (a) ITO/ MeO-2PACz/ perovskite/ PTAA/ Cu (reference) and (b) ITO/ MeO 2PACz/ DMPO4/ perovskite/ PTAA/ Cu (DMPO4). The trap-filled limit voltage (V_{TFL}) is extracted from the transition between the ohmic and trap-filled SCLC regions, yielding $V_{\mathrm{TFL}} = 0.54$ V for the reference device and $V_{\mathrm{TFL}} = 0.39$ V for the DMPO4-based device.

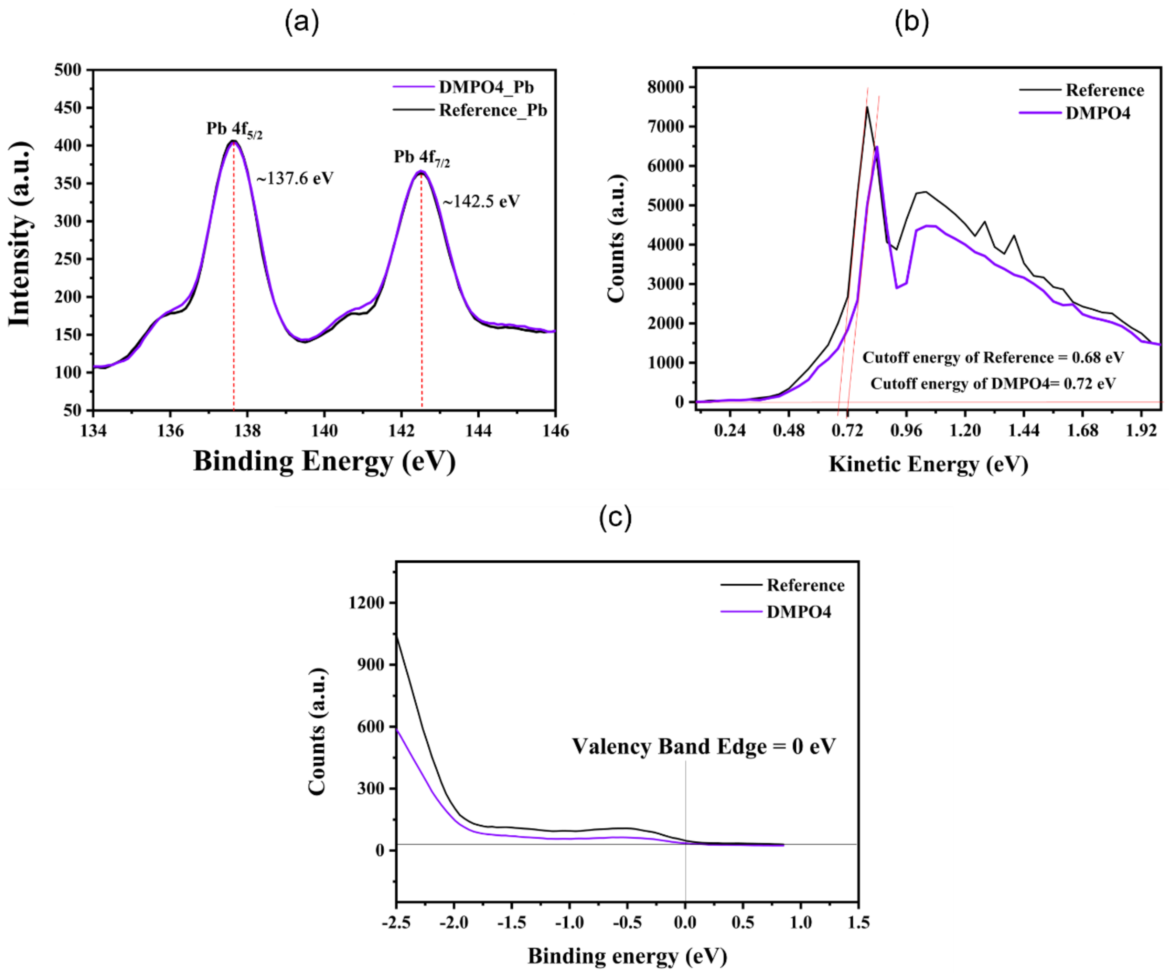

Figure S15: XPS and UPS spectra of Reference and DMPO4-interlayered perovskite films. (a) XPS spectra of Pb 4f orbitals; UPS spectra showing (b) secondary electron cutoff and (c) valence band edge (VBE).

From the UPS spectra, the work functions of the films were determined using the relation:

Work function = Fermi energy+ valence band edge+ cutoff energy

Work function of Reference = 4.07 +0+0.68= 4.75 eV

Work function of DMPO4 = 4.07+0+0.72= 4.79 eV.

For the reference perovskite film, $\Phi = 4.07 + 0 + 0.68 = 4.75$ eV, whereas the DMPO4-interlayered perovskite exhibits a slightly higher value of $\Phi = 4.07 + 0 + 0.72 = 4.79$ eV. These results are consistent with the Kelvin probe measurements, which yield work functions of 4.78 eV for the reference and 4.90 eV for the DMPO4-modified film. The small but noticeable increase in work function upon DMPO4 incorporation suggests a subtle modification of the surface potential, likely associated with interfacial dipole formation or improved surface passivation.

Bibliography

(1) Bannwarth, C.; Ehlert, S.; Grimme, S. GFN2-xTB—An Accurate and Broadly Parametrized Self-Consistent Tight-Binding Quantum Chemical Method with Multipole Electrostatics and Density-Dependent Dispersion Contributions. *J. Chem. Theory Comput.* **2019**, *15* (3), 1652–1671. https://doi.org/10.1021/acs.jctc.8b01176.

(2) Grimme, S. Exploration of Chemical Compound, Conformer, and Reaction Space with Meta-Dynamics Simulations Based on Tight-Binding Quantum Chemical Calculations. *J. Chem. Theory Comput.* **2019**, *15* (5), 2847–2862. https://doi.org/10.1021/acs.jctc.9b00143.

(3) Pracht, P.; Bohle, F.; Grimme, S. Automated Exploration of the Low-Energy Chemical Space with Fast Quantum Chemical Methods. *Phys. Chem. Chem. Phys.* **2020**, *22* (14), 7169–7192. https://doi.org/10.1039/C9CP06869D.

(4) Neese, F. The ORCA Program System. *WIREs Comput Mol Sci* **2012**, *2* (1), 73–78. https://doi.org/10.1002/wcms.81.

(5) Neese, F. Software Update: The ORCA Program System, Version 4.0. *WIREs Comput Mol Sci* **2018**, *8* (1). https://doi.org/10.1002/wcms.1327.

(6) Neese, F.; Wennmohs, F.; Becker, U.; Riplinger, C. The ORCA Quantum Chemistry Program Package. *J. Chem. Phys.* **2020**, *152* (22), 224108. https://doi.org/10.1063/5.0004608.

(7) Becke, A. D. Density-functional Thermochemistry. III. The Role of Exact Exchange. *The Journal of Chemical Physics* **1993**, *98* (7), 5648–5652. https://doi.org/10.1063/1.464913.

(8) Grimme, S.; Antony, J.; Ehrlich, S.; Krieg, H. A Consistent and Accurate *Ab Initio* Parametrization of Density Functional Dispersion Correction (DFT-D) for the 94 Elements H-Pu. *The Journal of Chemical Physics* **2010**, *132* (15), 154104. https://doi.org/10.1063/1.3382344.

(9) Schäfer, A.; Horn, H.; Ahlrichs, R. Fully Optimized Contracted Gaussian Basis Sets for Atoms Li to Kr. *The Journal of Chemical Physics* **1992**, *97* (4), 2571–2577. https://doi.org/10.1063/1.463096.

(10) Weigend, F.; Ahlrichs, R. Balanced Basis Sets of Split Valence, Triple Zeta Valence and Quadruple Zeta Valence Quality for H to Rn: Design and Assessment of Accuracy. *Phys. Chem. Chem. Phys.* **2005**, *7* (18), 3297–3305. https://doi.org/10.1039/B508541A.

(11) Zhang, Y.; Yang, W. Comment on ``Generalized Gradient Approximation Made Simple''. *Phys. Rev. Lett.* **1998**, *80* (4), 890–890. https://doi.org/10.1103/PhysRevLett.80.890.

(12) Zhao, Y.; Truhlar, D. G. The M06 Suite of Density Functionals for Main Group Thermochemistry, Thermochemical Kinetics, Noncovalent Interactions, Excited States, and Transition Elements: Two New Functionals and Systematic Testing of Four M06-Class Functionals

and 12 Other Functionals. *Theor Chem Account* **2008**, *120* (1–3), 215–241. https://doi.org/10.1007/s00214-007-0310-x.

(13) Yanai, T.; Tew, D. P.; Handy, N. C. A New Hybrid Exchange–Correlation Functional Using the Coulomb-Attenuating Method (CAM-B3LYP). *Chemical Physics Letters* **2004**, *393* (1), 51–57. https://doi.org/10.1016/j.cplett.2004.06.011.

(14) Chai, J.-D.; Head-Gordon, M. Systematic Optimization of Long-Range Corrected Hybrid Density Functionals. *The Journal of Chemical Physics* **2008**, *128* (8), 084106. https://doi.org/10.1063/1.2834918.

(15) Wittmann, L.; Neugebauer, H.; Grimme, S.; Bursch, M. Dispersion-Corrected r2SCAN Based Double-Hybrid Functionals. *J. Chem. Phys.* **2023**, *159* (22), 224103. https://doi.org/10.1063/5.0174988.

(16) Bannwarth, C.; Grimme, S. A Simplified Time-Dependent Density Functional Theory Approach for Electronic Ultraviolet and Circular Dichroism Spectra of Very Large Molecules. *Computational and Theoretical Chemistry* **2014**, *1040–1041*, 45–53. https://doi.org/10.1016/j.comptc.2014.02.023.

(17) Baik, M.-H.; Friesner, R. A. Computing Redox Potentials in Solution: Density Functional Theory as A Tool for Rational Design of Redox Agents. *J. Phys. Chem. A* **2002**, *106* (32), 7407–7412. https://doi.org/10.1021/jp025853n.

(18) Barone, V.; Cossi, M. Quantum Calculation of Molecular Energies and Energy Gradients in Solution by a Conductor Solvent Model. *J. Phys. Chem. A* **1998**, *102* (11), 1995–2001. https://doi.org/10.1021/jp9716997.

(19) Isegawa, M.; Neese, F.; Pantazis, D. A. Ionization Energies and Aqueous Redox Potentials of Organic Molecules: Comparison of DFT, Correlated Ab Initio Theory and Pair Natural Orbital Approaches. *J. Chem. Theory Comput.* **2016**, *12* (5), 2272–2284. https://doi.org/10.1021/acs.jctc.6b00252.
